# The statistical theory of linear selection indices from phenotypic to genomic selection

**DOI:** 10.1002/csc2.20676

**Published:** 2022-02-06

**Authors:** J. Jesus Cerón‐Rojas, Jose Crossa

**Affiliations:** ^1^ Biometrics and Statistics Unit, International Maize and Wheat Improvement Center (CIMMYT) Km 45 Carretera Mexico‐Veracruz, Edo. de Mexico Mexico DF CP 52640 Mexico

## Abstract

A linear selection index (LSI) can be a linear combination of phenotypic values, marker scores, and genomic estimated breeding values (GEBVs); phenotypic values and marker scores; or phenotypic values and GEBVs jointly. The main objective of the LSI is to predict the net genetic merit (*H*), which is a linear combination of unobservable individual traits’ breeding values, weighted by the trait economic values; thus, the target of LSI is not a parameter but rather the unobserved random *H* values. The LSI can be single‐stage or multi‐stage, where the latter are methods for selecting one or more individual traits available at different times or stages of development in both plants and animals. Likewise, LSIs can be either constrained or unconstrained. A constrained LSI imposes predetermined genetic gain on expected genetic gain per trait and includes the unconstrained LSI as particular cases. The main LSI parameters are the selection response, the expected genetic gain per trait, and its correlation with *H*. When the population mean is zero, the selection response and expected genetic gain per trait are, respectively, the conditional mean of *H* and the genotypic values, given the LSI values. The application of LSI theory is rapidly diversifying; however, because LSIs are based on the best linear predictor and on the canonical correlation theory, the LSI theory can be explained in a simple form. We provided a review of the statistical theory of the LSI from phenotypic to genomic selection showing their relationships, advantages, and limitations, which should allow breeders to use the LSI theory confidently in breeding programs.

## Core Ideas

- Linear selection indices (LSIs) are useful in plant and animal breeding.
- The main LSI objective is to predict the net genetic merit of individuals.
- We did a complete review of the statistical theory of LSIs.
- We described the bases of genomic LSIs.

**Abbreviations**
BLP: best linear predictor
BLUP: best linear unbiased predictor
BP: best predictor
CCA: canonical correlation analysis
CDMLPSI: constrained decorrelated multi‐stage linear phenotypic selection index
CLGSI: constrained linear genomic selection index
CLPSI: constrained linear phenotypic selection index
COMLPSI: constrained optimum multi‐stage linear phenotipic selection index
DMLPSI: decorrelated multi‐stage linear phenotypic selection index
ESIM: eigen selection index method
GEBV: genomic estimated breeding value
GESIM: genomic eigen selection index method
LGSI: linear genomic selection index
LMSI: linear molecular selection index
LPSI: linear phenotypic selection index
LSI: linear selection index
MESIM: molecular eigen selection index method
MSPE: minimum mean square prediction error
MVN: multivariate normal
OMLPSI: optimum multi‐stage linear phenotipic selection index
PPG‐ESIM: predetermined proportional gain eigen selection index method
QTL: quantitative trait loci
REML: restricted maximum likelihood
RESIM: restricted eigen selection index method
RL: rate of lay
RLPSI: restricted linear phenotypic selection index
SM: age at sexual maturity.

## 1 INTRODUCTION

Smith (1936) published a paper called “A discriminant function for plant selection,” in which he described a statistical method to select parents for the next selection cycle based on a linear combination of several joint quantitative traits. That method makes it possible to improve several traits that differ in additive variability, heritability, economic importance, and in the correlation among their phenotypes and genotypes in the plant and animal breeding context (Hazel et al., 1994). Hazel and Lush (1942) called that selection method “total score,” whereas Hazel (1943) called it “selection index,” and Cerón‐Rojas and Crossa (2018) called it “linear phenotypic selection index” (LPSI). Smith (1936) made a clear distinction between the LPSI (discriminant function) and the net genetic merit (*H*). The LPSI is a linear combination of observable phenotypic records, and *H* is an unobservable linear combination of trait breeding values (**g**) weighted by the trait economic values (**W**).

The main assumptions made by Smith (1936) to develop the LPSI theory are as follows: the vector of genotypic values **g** (the average of the phenotypic values across a [large] population of environments, or the genetic constitution of an organism or cell) is composed entirely of additive effects of genes and thus is the plant or animal breeding value, and *H* is the total individual genotypic value (Hazel & Lush, 1942; Kempthorne & Nordskog, 1959). Finally, the vectors of adjusted means of phenotypic values (**y**) and *H* have a joint multivariate normal distribution. By those assumptions, the joint distribution of *H* and LPSI is bivariate normal, whereas the conditional distribution of *H* given LPSI (and the conditional distribution of LPSI given *H*) is univariate normal. Under those assumptions, the regression of *H* on **y** is linear (Kempthorne & Nordskog, 1959), and it is possible to write the *H* and **y** values as a multiple linear regression model, where *H* and **y** are the dependent and independent variables, respectively, and both are random. In this model, the LPSI is the conditional expectation of *H* given **y**. Then, to predict *H*, one must find the values of the LPSI regression coefficients so that the LPSI values may best discriminate those individuals that have the highest *H* values (Smith, 1936).

Smith (1936) wrote his paper before the Eisenhart (1947) article, which highlighted that there are two fundamentally different explanatory variables, called “fixed” and “random” effects. One important result of this distintion is that whereas fixed effects influence only the mean of **y**, random effects influence only the variance of **y** and thus the LPSI and its parameters. Assuming that the population mean of *H* and **y** is zero, the selection response is the conditional expectation of *H* given the LPSI values, whereas the expected genetic gain per trait (or multi‐trait selection response) is the conditional expectation of **g** given the linear selection index (LSI) values (Cochran, 1951; Kempthorne & Nordskog, 1959; Smith, 1936). The estimates of these last parameters and the correlation between *H* and the LPSI are the main criteria to validate and compare the efficiency of any LSI.

Some authors (Finney, 1962; Hazel & Lush, 1942; Young, 1961) compared the LPSI efficiency with the independent culling method, where breeders sequentially select for a series of independent traits in the same population, and the tandem selection method, which involves sequential selection on a series of traits where repeated cycles of selection for a single trait are practiced until a desirable expression of the trait is reached. Those authors found that the LPSI selection response was higher than the selection response for the other two selection methods. Thus, under certain conditions (e.g., normality), the LPSI is the best option for selection.

**Core Ideas**

- Linear selection indices (LSIs) are useful in plant and animal breeding.
- The main LSI objective is to predict the net genetic merit of individuals.
- We did a complete review of the statistical theory of the LSI.
- We described the bases of genomic LSI.

The LPSI theory has been extended, and now a LSI can be a linear combination of marker scores (Lande & Thompson, 1990), genomic estimated breeding values (GEBVs) (Cerón‐Rojas & Crossa, 2019; Ceron‐Rojas et al., 2015), phenotypic values and marker scores (Lande & Thompson, 1990), and phenotypic values and GEBVs jointly (Dekkers, 2007). The last two indices are called “combined LSIs” in this review. An additional distinction is between single‐stage and multi‐stage LSIs (Cerón‐Rojas & Crossa, 2020a; Cerón‐Rojas et al., 2019a, 2019b; Cochran, 1951; Young, 1964; Xu & Muir, 1992). Multi‐stage LSIs are methods for selecting traits at different times or stages of the crop, such as the age at which breeders can measure and select the trait of economic interest and are used to save money and time because these indices do not need to have a large number of individuals in the selection process.

The main objectives of all LSIs are to predict *H* and select parents for the next generation; thus, the target of study of any LSI is not a parameter but rather the unobserved random *H* values. Additional objectives of LSIs are to maximize the selection response, the expected genetic gain per trait, and the correlation with *H*. By imposing restrictions equal to some predetermined values on the expected genetic gain per trait, several authors (Harville, 1975; Kempthorne & Nordskog, 1959; Mallard, 1972; Tallis, 1985) developed the constrained LPSI (CLPSI) theory. In addition, by applying the LPSI and CLPSI theories in the context of genomic selection, Ceron‐Rojas et al. (2015) and Cerón‐Rojas and Crossa (2019) developed the unconstrained and constrained linear genomic selection indices (LGSI and CLGSI, respectively), which are linear combinations of GEBVs. Cerón‐Rojas et al. (2016b) have shown that the CLPSI vector of coefficients is a linear combination of the LPSI vector of coefficients or a projection of the unconstrained LPSI vector of coefficients to a different space.

All the LSIs described above assume that the economic weights are known constants; however, for several reasons, this assumption is rarely fulfilled (Vandepitte & Hazel, 1977). Furthermore, several authors (Hazel, 1943; Lin, 1978; Yamada et al., 1975) have indicated that there is no one general method for assigning economic weights to the traits because those economic weights can change from time to time or vary from one location to another in breeding programs. For this reason, modified indices, such as the base index (Williams, 1962), modified base index (Smith, 1983), and non‐weighted multiplicative index (Elston, 1963), have been proposed in plant and animal breeding. However, those authors did not show that the latter LSIs maximize the correlation with the net genetic merit. In addition, it can be difficult to obtain the selection response and the expected genetic gain per trait for those indices.

The preceding problems led Cerón‐Rojas et al. (2008a, 2016a) to develop the eigen selection index method (ESIM), the restricted ESIM (RESIM), and the predetermined proportional gain ESIM (PPG‐ESIM) in the canonical correlation context (Hotelling, 1936). The ESIM is an unrestricted index, but the RESIM and PPG‐ESIM allow imposing null and predetermined restrictions, respectively, on the expected genetic gains of some traits, while the rest remain without restrictions. These indices do not require economic weights when predicting *H* and making selections because they use the first eigenvector of the matrix of multi‐trait heritabilities (in the ESIM case) or the first eigen vector of a linear transformation of the matrix of multi‐trait heritabilities (in the RESIM and PPG‐ESIM case) in the prediction. Cerón‐Rojas et al. (2008b) adapted the ESIM to the marker context and developed an index (molecular ESIM [MESIM]) similar to the linear molecular selection index (LMSI), whereas Cerón‐Rojas and Crossa (2018) adapted ESIM to the genomic selection context and developed a combined genomic eigen selection index method (genomic ESIM [GESIM]), similar to the Dekkers (2007) index.

All indices associated with the LPSI theory (unconstrained and constrained; phenotypic, genomic, multi‐stage, etc.) are particular cases of the best linear predictor (BLP) theory, which is based on the minimum mean square prediction error (MSPE), because this approach allows us to obtain the unique best MSPE predictor (Bickel & Doksum, 2015). The MSPE is the expected squared deviation between the true value and the estimate, and it provides an effective measure of proximity to the true parameter of interest (Carlin & Louis, 2000). On the other hand, all indices associated with ESIM are based on the canonical correlation analysis (CCA) theory (Hotelling, 1936).

Searle et al. (2006) have indicated that the best predictor (BP), BLP, and the best linear unbiased predictor (BLUP) are associated as follows. When all the parameters of the joint distribution of *H* and LSI are known, BP is available, whereas for BLP only the first and second moments are assumed to be known, and for BLUP the second but not first moments are assumed to be known. Under multivariate normality, BP and BLP are the same (Bickel & Doksum, 2015; Christensen, 2011; Harville, 2018); however, in practice, those approaches need estimates of their parameters. In this last case, the LSI could not be BLP (Searle et al., 2006). Nevertheless, with good estimates of the phenotypic and genotypic covariance matrices (e.g., by restricted maximum likelihood [REML] estimate), the BLP (or LPSI) is close to the BLUP (Christensen, 2011; Henderson, 1990).

Several authors (Baker, 1986; Bramscap, 1984; Dekkers & Settar, 2004; Hazel et al., 1994; Henderson, 1963; Lin, 1978; Moreau et al., 2007; Nordskog, 1978; Phillipsson et al., 1994) have given partial reviews of the LSIs theory, such as the unconstrained and constrained LPSI and the LMSI, among others. In addition, using minimum mathematical rigor, Cerón‐Rojas and Crossa (2018) wrote a user manual for breeders based on the LSI theory, and they gave many illustrative practical examples. In the current study, we reviewed the statistical theory of the LSIs from the unconstrained and constrained LPSI to the unconstrained and constrained LGSI. That is, based on the BLP theory, in the present work, we unified all the statistical theory of the LSIs associated with the LPSI (e.g., CLPSI, LGSI, CLGSI, etc.) from phenotypic to genomic selection. This requires greater mathematical rigor; as such, it is not possible to provide as many illustrative examples as Cerón‐Rojas and Crossa (2018) did. In addition, based on the CCA theory, we present a unified theory of the ESIM, RESIM, and PPG‐ESIM.

This review has four main parts. The first describes two earlier approaches to the unconstrained LSI theory and a description of the BLUP theory. The second describes the unconstrained LSI theory, including the LPSI, multi‐stage LPSI, LGSI, and combined LSI. Similarly, the third part contains the constrained LSI that includes the CLPSI, constrained multi‐stage LPSI, and CLGSI. Finally, in the fourth part, we describe ESIM, RESIM, PPG‐ESIM, and their relationships with the LPSI and CLPSI. The MESIM and GESIM are only variants of ESIM and the combined LSIs, which can be found in Cerón‐Rojas and Crossa (2018). The main objective of this review is to introduce researchers to the statistical theory of the LSIs. Our intended audience for this study is mainly plant breeders and graduate‐level students who are interested in LSI theory and who come from animal and plant breeding programs.

In subsection “Numerical example: LPSI vs ESIM,” we provide an illustrative example using the LPSI and ESIM theory. In subsection “RIndSel,” we introduce **RIndSel** (Alvarado et al., 2018; Pacheco et al., 2017; Perez‐Elizalde et al., 2014), an R‐software that is useful for estimating the LPSI, LGSI, CLPSI, ESIM, etc., parameters and for selecting individual candidates as parents for the next selection cycle. Furthermore, **RIndSel** is completely automated, which eliminates the need to learn a specialized syntax for anyone who is a novice user of R‐software. This means that the users only need to learn how to introduce their data into the program and how to interpret the **RIndSel** results. This software, and a complete **RIndSel** user manual, can be downloaded from https://data.cimmyt.org/dataset.xhtml?persistentId=hdl:11529/10854.

## 2 NOTATION

We use uppercase letters to denote random variables and lowercase letters to denote their particular realizations. For example, *X* is a random variable, whereas *x* (*X* = *x*) is a particular realization of *X*. Vectors are denoted with bold lowercase letters (e.g., **g** and **y**) and matrices with bold uppercase letters (**X**). Sometimes it is necessary to denote vectors with bold capital letters; for example, **E** denotes the vector of expected genetic gain per trait.

The expectation of the random variable *X*[*E*(*X*)] is scalar, whereas the conditional expectation of *X* given *Y* = *y* [*E*(*X*|*Y* = *y*)] is a function of *y*. Thus, before we observe *Y*, the value of *E*(*X*|*Y* = *y*) is unknown, so it is a random variable that we denote as *E*(*X*|*Y*).

## 3 TWO EARLY APPROACHES TO THE INDEX SELECTION THEORY

Pearson (1903) and Pearl and Surface (1909) were the first to describe the index theory before the Smith (1936) LPSI theory. As we shall see, Pearson (1903) predicted breeding values based on a linear mixed model, whereas Pearl and Surface (1909) used a nonlinear approximation to the index theory. In addition, whereas the Pearson (1903) index selects genotypes, the Pearl and Surface (1909) index selects phenotypes. This last index selects phenotypes because it does not maximize its correlation with the net genetic merit.

### 3.1 Pearson index

Suppose that **X** and **Z** are design matrices and that **θ** and **g** are vectors of fixed and random (e.g., breeding values) effects, respectively, associated to the trait of *n* individual candidates (**y**) to selection, and assume that **y** and **g** have multivariate normal (MVN) distribution, also denoted as “∼”, i.e., **y** ∼ MVN and **g** ∼ MVN. Consider the following linear mixed model:

(1) y=Xθ+Zg+ε

where **y** ∼ MVN(**Xθ**, **V**) is a *n* × 1 vector of observations for one trait, **Xθ** and V = σ*_g_* ^2^ **ZAZ**′ + **I** *_n_* σ_ε_^2^ are the unconditional expectation and variance of **y**, respectively, and var(**g**) = **A**σ*_g_* ^2^ is the unconditional variance of **g** ∼MVN (**0**, **A**σ*_g_* ^2^); var(**y**|**g**) = **I** *_n_* σ_ε_^2^ is the conditional variance of the vector of residuals **ε** ∼MVN (**0**, **I** *_n_* σ_ε_^2^), **0** denotes the null expectations of **g** and **ε**, **A** is the numerator relationship matrix (Lynch & Walsh, 1998; Mrode, 2014), and **I** *_n_* is an identity matrix of size *n* × *n*.

Based on the multivariate normal distribution theory, Pearson (1903) predicted **g** as

$$\hat{g}=E\left(g|y\right)=\sigma_{g}^{2}{\mathrm{AZ}}^{\prime }V^{-1}\left(y-X\hat{\theta }\right)$$

which now is called “single trait linear selection index” and where **V** ^−1^ is the inverse matrix of the variance of **y**, whereas $\hat{\theta }={\left(X^{\prime }V^{-1}X\right)}^{-1}X^{\prime }V^{-1}y$ (Xu, 2003). Henderson (1990) indicated that the above equation is one of the main Pearson (1903) results. Currently, $\hat{g}=E\left(g|y\right)$is called the BLUP of **g** and is one of the main tools to make genotypic selection in plant (Piepho et al., 2008) and animal (Mrode, 2014) breeding programs.

The BLUP properties have been developed by Henderson (1949, 1950, 1963, 1984, 1990). This author also showed that the BLUP and BLP (or LPSI) are similar; however, whereas the BLUP predicts vector **g**, the LPSI predicts a linear combination of **g** (i.e., the net genetic merit), as shown in Equations 2–4. In addition, Mrode (2014) indicated that BLUP reduces to LPSI when no adjustments for environmental factors are needed. This means that one of the main advantages of BLUP compared with BLP occurs when records have to be pre‐adjusted for fixed or for environmental effects. When the environmental effects are random, Xu (2003) writes Equation 1 as

$$y=1_{n}\mu +X\gamma +\mathrm{Zg}+W\pi +\varepsilon$$

where π is a vector of interaction genotypic–environment effects, **W** = **X** ⊗ **Z**, and ⊗ denotes the Kronecker product (Lych & Walsh, 1998). In this case, *E*(**y**) = **1** *_n_* μ, where **1** *_n_* is a vector of *n* ones, μ is the population mean, **V** = **X**var(**γ**)**X**′ + σ*_g_* ^2^ **ZAZ**′ + **W**var(π)**W**′ + **I** *_n_* σ_ε_^2^, and var(**γ**) and var(π) are the variance of **γ** and π, respectively. Note that in this last case, nongenetic effects (included in **γ**), such as location and year effects, are treated as random effects, whereas in Equation 1 those effects are treated as fixed and were included in **θ**. For additional details, see Xu (2003).

### 3.2 Pearl and Surface index

The Pearl and Surface (1909) approach to the index theory is based on the index numbers theory (Ralph et al., 2015). By this reasoning, those authors called to their index “selection index numbers.” The Pearl and Surface (1909) arguments to develop the selection index numbers are the same as those indicated by Smith (1936) in his paper. Pearl and Surface (1909) defined the selection index numbers as

$$I=\frac{{\upsilon^{\prime }}_{1}y}{{\upsilon^{\prime }}_{2}y^{*}}$$

where **y** is a vector of traits of economic interest for the breeder that become more desirable as their values increase, and y* is a vector of traits of economic interest that become more desirable as their values decrease. In the above equation, ***ν***_1_ and ***ν***_2_ are vector of constants to be given arbitrary values in the proportions that the different variables are to be weighted. The Pearl and Surface (1909) index can be considered a nonlinear selection index and is does not maximize its correlation with the net genetic merit (Equation 2). As we have indicated in the Introduction, in this work we describe the LSIs associated to the LPSI theory and the ESIM index theory.

### 3.3 BLUP in the multi‐trait context

Equation 1 is only useful to predict the individual breeding values associated to one trait. However, it is easy to extend Equation 1 to the multi‐trait selection context, and it is possible to include in Equation 1 non‐additive genetic effects, such as dominant genetic effects (or intra‐locus dominance allelic interaction) and epistasis effects (or inter‐loci allelic interaction). Multi‐trait evaluation is the optimum methodology to evaluate plants and animals because this method increases the accuracy of predictions when it accounts for the phenotypic and genetic correlation between individual traits (Mrode, 2014). For *t* traits, we can write the vector of breeding and phenotypic values as **g**′ = [**g**′_1_ **g**′_2_ … **g**′*_t_*] and **y**′ = [**y**′_1_ **y**′_2_ … **y**′*_t_*], respectively, from where, for two traits, Equation 1 can be written as

$$y_{1}=X_{1}\theta_{1}+Z_{1}g_{1}+\varepsilon_{1}$$

for Trait 1 and as

$$y_{2}=X_{2}\theta_{2}+Z_{2}g_{2}+\varepsilon_{2}$$

for Trait 2. Thus, in this context, Equation 1 is equivalent to the following model:

$$\left[\begin{array}{c} y_{1}\\ y_{2} \end{array}\right]=\left[\begin{array}{cc} X_{1} & 0\\ 0 & X_{2} \end{array}\right]\left[\begin{array}{c} \theta_{1}\\ \theta_{2} \end{array}\right]+\left[\begin{array}{cc} Z_{1} & 0\\ 0 & Z_{2} \end{array}\right]\left[\begin{array}{c} g_{1}\\ g_{2} \end{array}\right]+\left[\begin{array}{c} \varepsilon_{1}\\ \varepsilon_{2} \end{array}\right]$$

where **0** is a null matrix and the other parameters are defined in a similar manner as in Equation 1. Nevertheless, in this case $\mathrm{var}\left[\begin{array}{c} g_{1}\\ g_{2} \end{array}\right]=C⊗A$, where $C=\left[\begin{array}{cc} \sigma_{g_{1}}^{2} & \sigma_{g_{12}}\\ \sigma_{g_{21}} & \sigma_{g_{2}}^{2} \end{array}\right]$ is an additive genetic covariance matrix, and ⊗ and **A** are as defined in Equation 1. In addition, var(**y**_1_, **y**_2_ | **g**_1_, **g**_2_) = **R** ⊗ **I** *_n_*, where $R=\left[\begin{array}{cc} \sigma_{\varepsilon_{1}}^{2} & \sigma_{\varepsilon_{12}}\\ \sigma_{\varepsilon_{21}} & \sigma_{\varepsilon_{1}}^{2} \end{array}\right]$ is a residual covariance matrix. The unconditional covariance matrix of $\left[\begin{array}{cc} {y^{\prime }}_{1} & {y^{\prime }}_{2} \end{array}\right]$ is $V=\mathrm{var}\left[\begin{array}{c} y_{1}\\ y_{2} \end{array}\right]=Z^{*}\left[C⊗A\right]{Z^{\prime }}^{*}+R⊗I_{n}$, where $Z^{*}=\left[\begin{array}{cc} Z_{1} & 0\\ 0 & Z_{2} \end{array}\right]$, and **I** *_n_* is as defined in Equation 1. It is possible to extend the above equation to any number of traits of economic interest (Mrode, 2014).

For one trait, **g**_1_ can be partitioned into additive genetic (**a**_1_) effects (or intra‐locus additive allelic interaction), dominant genetic (**δ**_1_) effects, and epistasis (ι_1_) effects, such that **g**_1_ = **a**_1_ + δ_1_ + ι_1_. Epistasis refers to the interaction among additive and dominance genetic effects, for instance, additive by additive, dominance by dominance, additive by dominance, additive by additive by dominance, etc.; that is, ι_1_ = **aa**_1_ + **dd**_1_ + **ad**_1_ + …

When we separate non‐additive genetic effects from additive effects, we remove the confounding factors that would otherwise bias the results of the analysis (Cockerham, 1954; Fisher, 1918; Holland, 2001; Kempthorne, 1954; Mrode, 2014). In this case, the variance of **g**_1_ is $\mathrm{var}\left(g_{1}\right)=A\sigma_{a}^{2}+\Theta \sigma_{d}^{2}+\left(A\circ A\right)\sigma_{\mathrm{aa}}^{2}+\left(A\circ \Theta \right)\sigma_{\mathrm{ad}}^{2}+\left(\Theta \circ \Theta \right)\sigma_{\mathrm{dd}}^{2}+$ …

where Θ is the dominance relationship matrix, “∘” denotes the Hadamard product of two matrices or the element‐by‐element multiplication for unlinked loci (Gianola & de los Campos, 2009; Hill, 2010), σ_a_^2^ is the additive variance component, σ_d_^2^ is the deviation of dominance variance component, etc.

For *t* traits, asumming additive and dominance effects, and epistasis effects such as additive by additive, dominance by dominance, and additive by dominance, the variance of **g**′ = [**g**′_1_ **g**′_2_ … **g**′*_t_*] can be written as

$$\begin{array}{ccc} & & C⊗A+C_{D}⊗\Theta +C_{\mathrm{AA}}⊗\left(A\circ A\right)+C_{\mathrm{DD}}⊗\left(\Theta \circ \Theta \right)\\ & & \;+\;C_{\mathrm{AD}}⊗\left(A\circ \Theta \right) \end{array}$$

where **C** is as defined earlier; **C**_D_ is the covariance matrix of the dominant genetic interactions of effects between alleles within loci; **C**_AA_, **C**_DD_, and **C**_AD_ are the covariance matrices of the epistasis effects among loci; and **A**, Θ, ⊗, and “∘” are as defined above. Lynch and Walsh (1998) and Mrode (2014) have given details of how Equation 1 can be written for additive and non‐additive genetic effects jointly.

Although the above results seem very interesting, non‐additive effects are not transmitted to offspring in non‐inbreeding populations (Lynch & Walsh, 1998); the breeding value (or additive genetic effects **a**_1_) is the only component that can be selected and is thus the main component of interest in breeding programs (Mrode, 2014). Furthermore, in the index selection context, Andersson et al. (1998) found that the introduction of the dominance variance (e.g., 25% of the total phenotypic variance) has only a small positive effect (<1.5%) on the selection response; thus, to describe the LSI theory, the assumption that only additive effects are transmitted from generation to generation seems acceptable. This last asumption implies that the combined effects of all factors that make **y**′ = [**y**′_1_ **y**′_2_ … **y**′*_t_*] different from **g**′ = [**g**′_1_ **g**′_2_ … **g**′*_t_*], such as non‐additive effects, are in the residual vector (Hazel & Lush, 1942). Mrode (2014) indicated that non‐additive effects tend to be confounded with others, such as common maternal environment; thus, breeders should take care when they estimate non‐additive effects. Du and Hoeschele (2000) described methods to estimate additive and non‐additive variance components in the Bayesian context. In a similar manner, using linear mixed models theory, in the phenotypic and genomic selection context, Lynch and Walsh (1998) and Su et al. (2012), respectively, described methods to estimate additive and nonadditive variance components.

As we will see later, to predict *H*, the LPSI uses only matrix **C** and includes matrix **A** only for the estimation of **C** (Cerón‐Rojas & Crossa, 2018). The LPSI is the BLP of *H*, not the BLUP of *H* (Bulmer, 1980; Cochran, 1951; Henderson, 1963). However, as we have indicated in Equation 1, the BLP (LPSI) and BLUP theory are related, but, as we have indicated earlier, whereas the BLUP predicts **g**′ = [**g**′_1_ **g**′_2_ … **g**′*_t_*], the LPSI predicts linear combination of the elements of **g** (see Equations (2), (3), (4)).

### 3.4 Matrix **P** as a special case of matrix **V**

We have indicated that in the LSI context the assumption that only additive effects are transmitted from generation to generation is acceptable. For *t* traits, matrix **V** is equal to

$$\mathrm{var}\left[\begin{array}{c} y_{1}\\ \vdots \\ y_{t} \end{array}\right]=Z^{*}\left[C⊗A\right]{Z^{\prime }}^{*}+R⊗I_{n}=V$$

where

$$Z^{*}=\left[\begin{array}{c} \begin{array}{cc} Z_{1} & 0\\ 0 & Z_{2} \end{array}\begin{array}{cc} \text{…} & 0\\ \text{…} & \vdots \end{array}\\ \begin{array}{cc} \vdots & \vdots \\ 0 & \text{…}\;\; \end{array}\begin{array}{cc} \ddots & 0\\ 0 & Z_{t} \end{array} \end{array}\right]$$

whereas matrices **C** and **R** are of size *t* × *t*. Here, matrix **V** is associated with the prediction of vector **g**′ = [**g**_1_′ **g**_2_′ … **g** *_t_* ′] (Equation 1). Now suppose that matrix Z* = **I** *_nt_* (where **I** *_nt_* is an identity matrix of size *nt* × *nt*) and that the level of inbreeding among individuals is null; then **A** = **I** *_n_*, and the matrix **V** can be written as

V=C+R

As we shall see later, in this last case matrix **V** is associated with the BLP (LPSI) theory. Hereafter we denote it as **P**, which is the usual notation in the LPSI context.

By the foregoing results it is evident that, in the prediction of the net genetic merit (Equation 2), the LSI theory does not use matrix **A**. However, matrix **A** is used for the estimation of **C** (Cerón‐Rojas & Crossa, 2018). This seems to be a disaventage of the LPSI theory with respect to the BLUP theory. As we have indicated earlier, BLUP predicts vector **g**′ = [**g**_1_′ **g**_2_′ … **g** *_t_* ′], not the net genetic merit; this means that BLUP is associated only with a part of the LPSI theory: that associated to the expected genetic gain per trait (see Equations 8 and 10), which also predict the mean values of **g**′ = [**g**_1_′ **g**_2_′ … **g** *_t_* ′]. Jeyaruban et al. (1995, Abstract, p. 1) compared the efficiency of BLUP and the LPSI and concluded that: “The relative selection response with the selection indices compared to the BLUP estimates were from 94.5 to 99.4% of BLUP. Inbreeding was higher in the BLUP selected populations, which could offset any advantage of BLUP.”

## 4 UNCONSTRAINED LINEAR SELECTION INDICES

### 4.1 The LPSI theory

In this section, we describe the LPSI theory and all the LSIs associated to this theory.

### 4.2 Basic conditions to construct a valid LPSI in the single‐stage selection context

Conditions to construct a valid LPSI are as follows. (a) The phenotypic value is additively composed of two main parts: the genotypic value and the environmental contribution. (b) The genotypic value is the individual breeding value; thus, it is composed only of additive genes effects. (c) The net genetic merit of an individual is the genotypic economic value (Kempthorne & Nordskog, 1959). (d) Selection should be made at only one stage of the plant's or animal's life cycle. (e) The generations do not overlap. (f) All individuals below a certain level of desirability are culled without exception. (g) Selected individuals have an equal opportunity to have offspring (Hazel & Lush, 1942). (h) The LPSI values in the *c* ^th^ selection cycle and the LPSI values in the (*c* + 1)^th^ selection cycle are not correlated. (i) The correlation between the LPSI and the net genetic merit should be at its maximum in each selection cycle. These conditions are valid for all unconstrained or constrained LPSIs in the single‐stage selection context. Cerón‐Rojas and Crossa (2018) have given conditions for the unconstrained and constrained LGSI.

### 4.3 The net genetic merit

Let **g** *_q_* ′ = [*G_q_*_1_ *G_q_*_2_ … *G_qt_*] be a vector of true unobservable genotypic random variables for *t* traits with multivariate normal distribution and null expectation. Then the *q* ^th^ (*q* = 1, 2, …, *n*; *n *= number of individuals) individual net genetic merit is

(2) $$H_{q}=w^{\prime }g_{q}$$

where **w**′ = [*w*_1_ *w*_2_ … *w_t_*] is a 1 × *t* (*t* = number of traits) vector of known economic weights. The variance of *H_q_* is denoted as σ*_H_* ^2^ = **w**′**Cw**. Smith (1936) called Equation 2 “the genetic value of a plant or line.”

In Equation 2, **g**_q_′ = [*G_q_*_1_ *G_q_*_2_ … *G_qt_*] is a vector of random variables whereas in the BLUP equation described earlier, **g**′ = [**g**′_1_ **g**′_2_ … **g**′*_t_*] is a vector of particular realization of breeding values. Note that using the BLUP of **g**′ = [**g**′_1_ **g**′_2_ … **g**′*_t_*] (i.e., ${\hat{g}}^{\prime }=\left[\begin{array}{cccc} {{\hat{g}}^{\prime }}_{1} & {{\hat{g}}^{\prime }}_{2} & \text{…} & {{\hat{g}}^{\prime }}_{t} \end{array}\right]$), a predictor of Equation 2 is:

$$\hat{h}=\sum_{j=1}^{t}w_{j}{\hat{g}}_{j}$$

where ${\hat{h}}^{\prime }=\left[\begin{array}{cccc} {\hat{H}}_{1} & {\hat{H}}_{2} & \text{…} & {\hat{H}}_{n} \end{array}\right]$ is a vector of estimated net genetic merit values for *n* indviduals, and *w_j_* is the *j^th^* element of the vector of economic weights **w** (Dekkers, 1997; Portes et al., 2020). The predictor $\hat{h}$ combines the BLUP and LPSI theory to predict the net genetic merit.

### 4.4 The net genetic merit and its relationship with **y**

For the *q^th^* individual, the relationship between Equation 2 and the vector of random phenotypic variables **y**′*_q_* = [*Y_q_*_1_ *Y_q_*_2_ … *Y_qt_*] for *t* traits is

(3) $$H_{q}=m_{H}+\beta^{\prime }\left(y_{q}-\mu \right)+e_{q}$$

where *m_H_* is the mean of *H_q_*, **μ**′ = [μ_1_ μ_2_ … μ*_t_*] is a vector 1 × *t* of phenotypic means of **y** *_q_*, and *e_q_* is the deviation representing the extent to which **β**′(**y** *_q_* − **μ**) differs from the expected value of *H_q_* (Young, 1964). In Equation 3, **y**′*_q_* = [*Y_q_*_1_ *Y_q_*_2_ … *Y_qt_*] is a vector of random variables, whereas in the BLUP equation described earlier, **y**′ = [**y**′_1_ ***y*** ′_2_ … **y**′*_t_*] is a vector of particular realization of phenotypes values for *t* traits. In addition, the *j^th^* (*j *= 1, 2, …, *t*) element of vector **μ** is equivalent to **Xθ** (Equation 1) when **X** = **1** (a vector of 1s) and **θ** = μ*_j_*. We have assumed that *e_q_* has a normal distribution, null expectation, and variance σ*_e_* ^2^ and that the covariance between any pairs of *e_q_* and *e_r_* (*q* ≠ *r*) is zero. As we will see later, **β**′(**y** *_q_* − **μ**) = *I_q_* is the LPSI. It is possible to show that the random error (*e_q_*) and the LPSI are uncorrelated (Bickel & Doksum, 2015). The variance of *H_q_* can be written as σ*_H_* ^2^ = **w**′**Cw** = σ*_I_* ^2^ + σ*_e_* ^2^, where σ*_I_* ^2^ = **β**′P**β** is the variance of *I_q_*, and **P** = **C** + **R** is the phenotypic covariance matrix of **y**′*_q_* = [*Y_q_*_1_ *Y_q_*_2_ … *Y_qt_*]; **C** is as defined earlier, and **R** is the residual covariance matrix. All these last three matrices are of size *t* × *t*.

Equation 3 is a multiple linear regression model where the independent (**y** *_q_*) and dependent (*H_q_*) variables are random and have a joint multivariable normal distribution (Rencher & Schaalje, 2008). Nevertheless, whereas in the standard regression model the values of *H_q_* and **y** *_q_* are observables, in Equation 3 only the **y** *_q_* values are observable. For this reason, *H_q_* should be predicted using, for example, the BLP.

### 4.5 The BLP of *H*

Hereafter we will denote the vector of phenotypic trait random variables as **Y** and its particular realization values as **y**; and, to simplify notation, we will denote *H_q_* and *I_q_* as *H* and *I*, respectively. Suppose that *H* and **Y** have a joint multivariate normal distribution and that we are looking for a linear predictor of *H* assuming that **Y** is the only information that we can use to predict the unobsevable random variable *H*. We want to find a predictor function of **Y** such that this predictor is “close” to *H* in the sense of the MSPE, which is a measure used in the mathematical theory of prediction (Bickel, 2015). Suppose that *E*(*H*) = *m_H_*, *E*(**Y**) = **μ**, and that var(*H*), var(**Y**) = **P**, and the covariance between *H* and **Y** [cov(*H*, **Y**) = **w**′**C**] exist and are finite, then the BLP of *H* (or the LPSI) is

(4) $$E\left(H|Y=y\right)=m_{H}+w^{\prime }CP^{-1}\left(y-\mu \right)=\beta_{0}+\beta^{\prime }y$$

where β_0_ = *m_H_* − **β**′**μ**, **w**′**C** and **μ** are as defined above, **P** ^−1^ is the inverse of the matrix **P**, and **β** = **P** ^−1^ **Cw**. Cochran (1951) was the first to indicate that the LPSI is the BLP of *H*. Equation 4 is unique and unbiased (Christensen, 2011; Searle et al., 2006) and represents a model that is linear in **y** as well as in **β**. In addition, it indicates that the problem of predicting *H* is simply estimating its conditional mean, whereas the LPSI variance is σ*_I_* ^2^ = **β**′**P** **β**. The assumption of multivariate normal distribution of *H* and **Y** is a sufficient condition for Equation 4 to be linear; moreover, any function that is expressible as Equation 4 is regarded as linear even if the vector **β** depends on the joint distribution of *H* and **Y** (Bickel & Doksum, 2015; Harville, 2018). Smith (1936) called Equation 4 “discriminant function.”

By Equations (1), (2), (3), (4), the random vectors **g** and **Y** have joint multivariate normal distribution with mean *E*(**g**) = **0** and *E*(**Y**) = **μ**, and covariance matrix $\mathrm{var}\left(\begin{array}{c} Y\\ g \end{array}\right)=\left[\begin{array}{cc} P & C\\ C & C \end{array}\right]$, where **P** and **C** are as defined above. Moreover, the distribution of LPSI and *H* = **w**′**g** values is a bivariate normal distribution, with mean *E*(*H*) = *m_H_* and *E*(*I*) = *m_I_* and covariance matrix $\mathrm{var}\left[\begin{array}{c} H\\ I \end{array}\right]=\left[\begin{array}{cc} w^{\prime }\mathrm{Cw}\; & w^{\prime }C\beta \\ w^{\prime }C\beta \; & \beta^{\prime }P\beta \end{array}\right]=\left[\begin{array}{cc} \sigma_{H}^{2}\; & \sigma_{\mathrm{HI}}\\ \sigma_{\mathrm{HI}}\; & \sigma_{I}^{2} \end{array}\right]$, where **β**, **w**′, σ*_H_* ^2^, and σ*_I_* ^2^ were defined earlier and σ*_HI_* = **w**′**C**β is the covariance between *H* and LPSI.

As we will see later, because we can assume that β_0_ = *m_H_* − **β**′**μ** is a fixed contant, the maximized LPSI expected genetic gain per trait (Equation 10), the maximized correlation between *H* and LPSI (Equation 11), and the maximized LPSI selection response (Equation 12), are not affected if in Equation 4 we assume that *E*(*H*) = 0 and *E*(**Y**) = **0**. Likewise, when we select parents for the next generation, they will be the same in both cases, that is, when *E*(*H*) = 0 and *E*(**Y**) = **0** and when *E*(*H*) = *m_H_* and *E*(**Y**) = **μ**. Thus, we think that there are not loss of generality or loss of accuracy to describe the LSI theory if we assume that *E*(*H*) = 0 and *E*(**Y**) = **0**. In practice, *E*(**Y**) = **0** only if **y** is centered with respect to **μ**.

### 4.6 The LPSI prediction error

Because **β** = **P** ^−1^ **Cw**, then σ*_HI_* = **w**′**Cβ** and σ*_I_* ^2^ = **β**′**P** **β** are the same, from where the variance of the LPSI prediction error is

(5) $$\mathrm{var}\left(H|I\right)=\sigma_{H}^{2}-\frac{\sigma_{HI}^{2}}{\sigma_{I}^{2}}=\sigma_{H}^{2}-\sigma_{I}^{2}=\sigma_{e}^{2}$$

Equation 5 and equation σ*_e_* ^2^ = σ*_H_* ^2^(1 − ρ*_HI_* ^2^) are the same, where ρ*_HI_* ^2^ = (σ*_HI_* ^2^)/(σ*_H_* ^2^σ*_I_* ^2^) and σ*_HI_* ^2^ = (**w**′**C**β)^2^ are, respectively, the square of correlation and the square of covariance between *H* and LPSI. We defined the other parameters earlier.

The conditional mean of *H* is dependent on **y** (Equation 4); however, the conditional variance of *H* (Equation 5) is independent of **y**. Thus, under the multivariate normal assumption, Equations 4 and 5 provide a linear model with constant variance (Rencher & Schaalje, 2008). Moreover, if the marginal density of LPSI is normal and if the conditional density of *H* given LPSI is normal for every LPSI value, the assumption of bivariate normality of LPSI and *H* is guaranteed (Arnold et al., 1999; Kagan & Wesolowski, 1996).

### 4.7 The intensity of selection in the single‐stage context

Let *I* be transformed into a random variable *T*, as *T* = (*I* − *m_I_*)/σ*_I_*, where *E*(*I*) = *m_I_* and σ_1_ is the standard deviation of *I*. Assume that all *I* values higher than *I* ^*^value corresponding to any proportion of selected individuals (*p*) will be selected; then the variables T* = (I* − *m_I_*)/σ*_I_* and *T* are related to selection intensity (*k*) as

(6) $$k=\frac{1}{p}E\left(T\right)=\frac{1}{p}\int_{\tau^{*}}^{\infty }\frac{\tau }{\sqrt{2\pi }}\exp\left\{-\frac{1}{2}\tau^{2}\right\}d\tau =\frac{z(\tau^{*})}{p}$$

where $z\left(\tau^{*}\right)=\frac{\exp\{-0.5\tau^{*2}\}}{\sqrt{2\pi }}$ is the height of the ordinate of the normal curve at the lowest value of τ* retained, and *p* is the proportion of the population of animal or plant lines that will be selected (Smith, 1936). In the selected population, Equation 6 is the uncoditional expectation of the random variable *T* [*E*(*T*)] weighted by *p* and was obtained by Smith (1936). It is possible to show that the variance of *T* (weighted by *p*), over the interval (τ*, ∞), is equal to 1 + *k*(τ* − *k*). Several authors (Hazel & Lush, 1942; Kempthorne & Nordskog, 1959; Xu & Muir, 1992; Young, 1964) have given additional details of Equation 6.

### 4.8 The conditional expectation of *H* and **g** given *I*

Suppose that the expectations of *H* and **g** are null, whereas the expectation of *I* is *E*(*I*) = *m_I_*; then the conditional expectation of *H* and **g** given *I* is, respectively,

(7) $$E\left(H|I\right)=\frac{\mathrm{Cov}(H,I)}{\sigma_{I}^{2}}\left(I-m_{I}\right)=\frac{\beta^{\prime }Cw}{\sigma_{I}}\frac{I-m_{I}}{\sigma_{I}}=\frac{\beta^{\prime }Cw}{\sigma_{I}}T$$

and

(8) $$E\left(g|I\right)=\frac{\mathrm{Cov}(g,I)}{\sigma_{I}^{2}}\left(I-m_{I}\right)=\frac{C\beta }{\sigma_{I}}\frac{\left(I-m_{I}\right)}{\sigma_{I}}=\frac{C\beta }{\sigma_{I}}T$$

where cov(*H*, *I*) = **β**′**Cw** is the covariance between *H* and *I*, cov(**g**, *I*) = **C** **β** is the covariance between **g** and *I*, *T* = (*I* − *m_I_*)/σ*_I_*, and σ*_I_* ^2^ = **β**′**P** **β** and σ_1_ are the variance and standard deviations of *I*, respectively. In Equation 7, cov(*H*, *I*)/σ*_I_* ^2^ is the relationship between *E*(*H*|*I*) and *I* (or the regression of *H* on *I*), and cov(**g**, *I*)/σ*_I_* ^2^ is the relationship between *E*(**g**|*I*) and *I* in Equation 8.

### 4.9 The LPSI selection response, expected genetic gain per trait, and correlation

By Equations (5), (6), (7), the LPSI selection response (*R*) and expected genetic gain per trait (**E**) are, respectively,

(9) $$R=k\frac{\beta^{\prime }Cw}{\sigma_{I}}=k\sigma_{H}\rho_{\mathrm{HI}}$$

and

(10) $$E=k\frac{C\beta }{\sigma_{I}}$$

where *k* was defined in Equation 6, $\sigma_{H}=\sqrt{w^{\prime }Cw}$ is the standard deviation of *H*, and ρ*_HI_* = σ_HI_/σ*_H_* σ*_I_* is the correlation between *H* and LPSI. All the other parameters of Equations 9 and 10 were defined earlier. Smith (1936) obtained Equation 9, and Hanson and Johnson (1957) were the first to describe Equation 10. In Equation 9, *R* is a scalar; in Equation 10, **E** is a vector *t* × 1 (*t* = number of traits) of the expected genetic gain value of each trait. In addition, *R* is the expectation of *H*, and **E** is the expectation of **g**.

To maximize the selection response (Equation 9), Smith (1936) used differential calculus to derive Equation 9 with respect to **β**. The result reported by this author was the vector of regression coefficients **β** = **P** ^−1^ **Cw**. He indicated that **β** = **P** ^−1^ **Cw** should maximize the selection response. Thus, Smith (1936) did not use the BLP theory to develop the LPSI theory because using differential calculus is not necessary to obtain **β** = **P** ^−1^ **Cw** in the BLP context (Equation 4).

### 4.10 The maximized correlation between *H* and *I*

Because **β** = **P** ^−1^ **Cw**, the maximized correlation between *H* and *I* is

(11) $$\rho_{\max}=\frac{\sqrt{\beta 'P\beta }}{\sqrt{w'\mathrm{Cw}}}=\frac{\sqrt{w'CP^{-1}\mathrm{Cw}}}{\sqrt{w'Cw}}$$

where all the terms were defined earlier. Equation 11 represents the proportion of the variance of *H* that can be attributed to the regression relationship with *I*. The covariance (σ*_HI_*) and correlation are good measures of relationship only for variables with linear trends and are generally unsuitable for non‐normal random variables with a curvilinear relationship (Rencher, 2002). That is, Equation 11 is a measure of linear dependency between *H* and *I* and is called the “multiple correlation coefficient,” whereas $\rho_{HI_{\max}}^{2}=\frac{\sigma_{I}^{2}}{\sigma_{H}^{2}}$ is called “the coefficient of determination or squared multiple correlation” (Rencher & Schaalje, 2008). Note that while ρ*_HI_* = σ*_HI_*/σ*_H_* σ*_I_* can take any value in the interval −1.0 and 1.0, Equation 11 gives the maximum value of ρ*_HI_*, and when ρ*_HI_* = 0, *H* and *I* are independent.

### 4.11 The maximized selection response

Once again, because **β** = **P** ^−1^ **Cw**, the maximized LPSI selection response is

(12) $$R_{\max}=k\sqrt{\beta 'P\beta }=k\sqrt{w'CP^{-1}Cw}$$

Equation 12 predicts the mean improvement in *H* attributable to indirect selection on *I* only when **β** = **P** ^−1^ **Cw** and is proportional to $\sqrt{\beta 'P\beta }$ and *k*. Thus, whereas in Equation 9 the selection response can take any value, Equation 12 gives the maximum value of Equation 9. This is the main difference between the two equations.

Cerón‐Rojas et al. (2006) showed that Equation 12 is related to the Cauchy–Schwarz inequality, which corroborates that **β** = **P** ^−1^ **Cw** is a global minimum when the mean squared difference between *I* and *H*, *E*[(*H* − *I*)^2^], is minimized, and a global maximum when the ρ*_HI_* correlation between *I* and *H* is maximized because Equation 12 is true only when **β** = **P** ^−1^ **Cw**.

### 4.12 The maximized correlation, selection response, and expected genetic gains have the same form for all LSIs

Equations 9–12 have the same form for all LSIs described in this review. Thus, as we will see later, the only difference between those equations and the selection response, expected genetic gain per trait, and correlation, associated with the other LSIs, is the type of information that the LSI vector of coefficients and the matrices contain.

### 4.13 Summary of the LPSI statistical properties

When *H* and *I* have a joint bivariate normal distribution; **β** = **P** ^−1^ **Cw**; and **P**, **C**, and **w** are known, the statistical LPSI properties are the following:

1. Equations 10–12 are maximum.
2. The variance of *I* and the covariance between *H* and *I* are the same.
3. The maximized correlation between *H* and *I* is equal to Equation 11.
4. The LPSI is unbiased in the sense that *E*(*I*) = *E*(*H*) (Searle et al., 2006).
5. The variance of the predicted error (Equation 5) is minimal.
6. The total variance of *H* explained by *I* is σ*_I_* ^2^ = ρ*_HI_* ^2^σ*_H_* ^2^.
7. Selecting any upper fraction of the population based on values of *I* (Equation 4) ensures that for that selected proportion, *E*(*H*) is maximized (Equation 9). That is, with truncation selection, Equation 4 maximizes the expected mean of the selected individuals (Cochran, 1951; Henderson, 1990).
8. The *I* values are appropriate to make selection, i.e., selecting the plants or animals with the highest predictions to be parents of the next generation (Searle et al., 2006).
9. The LPSI is the BLP of *H* in the sense that it (a) is a linear function of **y**; (b) is “best” in the sense that it has minimum mean squared error of prediction; and (c) is optimal and unique among the class of linear predictors based on **y** (Robinson, 1991; Searle et al., 2006).

Cerón‐Rojas and Crossa (2018) showed that all the LSIs associated with the LPSI have similar properties as those described in Points 1–9, as exemplified by CLPSI and CLGSI. This occur because the CLPSI and CLGSI vector of coefficients are linear combinations of the LPSI and LGSI vector of coefficients or projections of these vectors to a different space.

### 4.14 Multi‐stage LPSIs

One stage is the animal or plant age at which breeders can measure and select the trait of economic interest. Cochran (1951) and Young (1964) combined the LPSI theory with the independent culling method and developed the optimum multi‐stage LPSI (OMLPSI), which can include several stages in each selection cycle. The OMLPSI takes into consideration the correlation among indices at different stages when making selections and requires multiple integration techniques to derive selection intensities. In addition, after the first selection stage, the OMLPSI values could be non‐normally distributed, and there are problems of convergence when the traits and the index values are highly correlated (Börner & Reinsch, 2012).

Xu and Muir (1992) developed the decorrelated multi‐stage LPSI (DMLPSI) as one possible solution to the OMLPSI problems. After the first selection stage, the DMLPSI values could be normally distributed, and this index does not require multiple integration techniques to derive selection intensities because it maximizes the correlation between the index and the net genetic merit at each stage under the restriction that the covariance between the DMLPSI values at different stages be zero. To obtain truncation points and selection intensities, Xu and Muir (1992) derived a set of nonlinear equations that can be solved iteratively using a multidimensional Newton method. One problem associated with DMLPSI is that the selection responses and correlation with *H* are lower than the OMLPSI selection response and correlation after the first selection stage (Cerón‐Rojas et al., 2019a, 2019b).

### 4.15 The OMLPSI

As we have indicated earlier, there is no loss of generality if we assume that *E*(*H*) = 0 and *E*(**y**) = **0** to describe the LSI theory. Thus, to simplify notation, hereafter we will assume that *E*(*H*) = 0 and *E*(**y**) = **0**. These last two assumptions are common in the multi‐stage LPSI context (Xie & Xu, 1997, 1998; Xu & Muir, 1992; Young, 1964). In practice, *E*(**y**) = **0** only if **y** is centered with respect to **μ** = *E*(**y**).

Let **y**′ = [*Y*_1_ *Y*_2_ … *Y_t_*] be a vector of random variables for *t* traits. We can partition this vector into *S* (*t_i_* < *S* < *t*, where *t_i_* is the number of traits at Stage *i*) subvectors as **y**′ = [**ω**′_1_ **ω**′_2_ … **ω**′*_S_*], where ${\omega^{\prime }}_{i}=\left[\begin{array}{cccc} Y_{1} & Y_{2} & \text{…} & Y_{t_{i}} \end{array}\right]$ is a subvector of **y** at Stage *i*. Thus, at Stage S, $t=\sum_{i=1}^{S}t_{i}$ is the total number of selected traits from vector **y** and, for each stage, the BLP (or OMLPSI) of *H* is

(13) $$\begin{array}{ccc} E(H|\omega_{1}) & = & {\beta^{\prime }}_{1}\omega_{1}\;,\;E\left(H|\omega_{1},\omega_{2}\right)={\beta^{\prime }}_{2}\omega_{2}^{*},\;\text{…},\;\\ & & E\left(H|\omega_{1},\omega_{2},\text{…},\omega_{S}\right)={\beta^{\prime }}_{S}y \end{array}$$

where **ω**′*_2_ = [**ω**′_1_ **ω**′_2_]. In Equation 13, the BLP changes at each stage; however, the form of this predictor is the same as that of the LPSI.

At Stage *i*, let *I_i_* = **β**′*_i_* **ω** *_i_* be the index, where $\beta_{i}^{\prime }=\left[\begin{array}{cc} \beta_{i1} & \beta_{i2} \end{array}\;\begin{array}{cc} \text{…} & \beta_{it_{i}} \end{array}\right]$ is the OMLPSI vector of coefficients, and **ω** *_i_* and *t_i_* are as defined earlier; then we can construct the OMLPSIs as follows:

$$\left[\begin{array}{c} I_{1}\\ I_{2}\\ \begin{array}{c} \vdots \\ I_{S} \end{array} \end{array}\right]=\;\left[\begin{array}{c} \begin{array}{cc} \beta_{1}^{\prime } & 0\\ \beta_{1}^{{}^{\prime }} & \beta_{2\;}^{{}^{\prime }} \end{array}\begin{array}{cc} \text{…} & 0\\ \text{…} & 0 \end{array}\\ \begin{array}{cc} \vdots & \vdots \\ \beta_{1}^{{}^{\prime }} & \beta_{2}^{\prime } \end{array}\begin{array}{cc} \ddots & \vdots \\ \text{…} & \beta_{S}^{\prime } \end{array} \end{array}\right]\;\left[\begin{array}{c} \omega_{1}\\ \omega_{2}\\ \begin{array}{c} \vdots \\ \omega_{S} \end{array} \end{array}\right]$$

This last result indicates that until Stage *S* − 1, each index is partial, but at Stage *S*, *I_S_* = **β**′_1_ω_1_ + **β**′_2_ **ω**_2_ + … + **β**′*_S_* **ω** *_S_* is a whole index. Young (1964) called to this procedure “the part and whole index selection,” whereas Xu and Muir (1992) called it “selection index updating” because as traits become available, each subsequent index contains all traits available up to that current stage. The above equation is valid for any unconstrained or constrained multi‐stage index.

### 4.16 The OMLPSI vector of coefficients

Let **P** = {**P** *_ij_*} be the covariance matrix of vector **y**′ =[**ω**′_1_ **ω**′_2_ … **ω**′*_S_*], where **P** *_ij_* = cov(**ω** *_i_*, **ω** *_j_*) is the *ij* ^th^ (*i*, *j* = 1, 2, …, *S*) submatrix of **P**, and let **Λ** = {cov(**ω** *_i_*, **g**)}′ = [**C**_1_ **C**_2_ … **C** *_S_*] be the covariance matrix between **ω** *_i_* (*i* = 1, 2, …, *S*) and **g**, where cov(**ω** *_i_*, **g**) = **C** *_i_* is a submatrix of matrix **Λ** at Stage *i*. Moreover, let $Q_{\mathrm{ii}}$ and ${\Lambda^{\prime }}_{i}=\left[C_{1}\;C_{2}\;\text{…}\;C_{i}\right]$ be submatrices of **P** and **Λ** until Stage *i* (Cerón‐Rojas et al. 2019a), respectively, then the OMLPSI vector of coefficients that maximizes Equations 9 and 10 (and the correlation between OMLPSI and *H*) at Stage *i* is

(14) $$\beta_{i}=Q_{ii}^{-1}\Lambda_{i}w$$

where **Q** *_ii_* ^−1^ is the inverse of matrix **Q** *_ii_*, and **w**′ = [*w*_1_ *w*_2_ … *w*_t_] is as defined earlier.

### 4.17 Maximized selection response, expected genetic gain per trait, and correlation at Stage *i*

By Equation 14, the maximized selection response ($R_{\max_{i}}$), expected genetic per trait ($E_{\max_{i}}$), and correlation ($\rho_{\mathrm{ma}x_{i}}$) at Stage *i*, respectively, are

(15) $$\begin{array}{ccc} R_{\max_{i}} & = & k_{i}\sqrt{{\beta^{\prime }}_{i}Q_{\mathrm{ii}}\beta_{i}},\;E_{\max_{i}}=k_{i}\frac{\Lambda_{i}\beta_{i}}{\sqrt{{\beta^{\prime }}_{i}Q_{\mathrm{ii}}\beta_{i}}},\;\mathrm{and}\;\rho_{\mathrm{ma}x_{i}}\\ & = & \frac{\sqrt{{\beta^{\prime }}_{i}Q_{\mathrm{ii}}\beta_{i}}}{\sqrt{w^{\prime }Cw}} \end{array}$$

where *k_i_* is the selection intensity. For all stages, the total selection response and expected genetic gain per trait are $R_{T}=\sum_{i=1}^{S}R_{\max_{i}}$ and $E_{T}=\sum_{i=1}^{S}E_{\max_{i}}$, respectively.

### 4.18 DMLPSI

#### 4.18.1 Covariance matrix to make the DMLPSI values among stages null

Let **ω**′_(_ *_i_*_−1)_ = [**ω**′_1_ **ω**′_2_ … **ω**′*_i_*_−1_] and **ω**′_(_ *_i_*_)_ = [**ω**′_1_ **ω**′_2_ … **ω**′*_i_*] (where ${\omega^{\prime }}_{i}=\left[\begin{array}{cccc} Y_{1} & Y_{2} & \text{…} & Y_{t_{i}} \end{array}\right]$ is a subvector of **y**′ = [*Y*_1_ *Y*_2_ … *Y_t_*] at Stage *i*) be subvectors of **y** until Stages *i* − 1 and *i*, and let *t_i_*_−1_ and *t_i_*_−1_ + *t_i_* be the size of vectors **ω**′_(_ *_i_*_−1)_ and **ω**′_(_ *_i_*_)_, respectively; then the covariance matrix between **ω**′_(_ *_i_*_−1)_ and **ω**′_(_ *_i_*_)_ (**Q**_(_ *_i_*_−1)_ ***_i_***) will be of size *t_i_*_−1_ × (*t_i_*_−1_ + *t_i_*) and can be written as

$$\;Q_{\left(i-1\right)i}=\;\left[\begin{array}{cccc} \sigma_{11} & \sigma_{12} & \text{…} & \sigma_{1(n_{i-1)}}\\ \sigma_{21} & \sigma_{22} & \text{…} & \sigma_{2(n_{i-1)}}\\ \vdots & \vdots & \ddots & \vdots \\ \sigma_{(n_{i-1})1} & \sigma_{(n_{i-1})2} & \text{…} & \sigma_{\left(n_{i-1}\right)\left(n_{i-1}\right)} \end{array}\;\;\;\;\begin{array}{c} \sigma_{1\left(n_{i-1}+n_{i}\right)}\\ \sigma_{1\left(n_{i-1}+n_{i}\right)}\\ \vdots \\ \sigma_{\left(n_{i-1}\right)\left(n_{i-1}+n_{i}\right)} \end{array}\right]$$

Matrix **Q**_(_ *_i_*_–1)_ *_i_* is a nonsquare and nonsymmetric phenotypic variance matrix and is useful by making the DMLPSI and constrained decorrelated multi‐stage LPSI (CDMLPSI) values independent among stages. By definition, a covariance matrix is symmetric; however, in this case, matrix **Q**_(_ *_i_*_–1)_ *_i_* is of size *t_i_*_−1_ × (*t_i_*_−1_ + *t_i_*) because until Stages *i* − 1 there are *t_i_*_−1_ traits, whereas at Stage *i* there are *t_i_*_−1_ + *t_i_* traits. This means that at Stage *i* − 1 we have an index with *t_i_*_−1_ traits, whereas at Stage *i* we have an index with *t_i_*_–1_ + *t_i_*; that is, the trait selected until Stage *i* − 1 and the traits selected until Stage *i*. By this reasoning, as we indicated earlier, Young (1964) called this procedure “the part and whole index selection,” whereas Xu and Muir (1992) called it “selection index updating” because as traits become available, each subsequent index contains all traits available up to that current stage.

#### 4.18.2 The DMLPSI vector of coefficients at Stage *i*

Let *I_i_*_−1_ = **b**′*_i_*_−1_ **ω** *_i_*_−1_ and *I_i_* = **b**′*_i_* **ω** *_i_* be the DMLPSIs at Stages *i* – 1 and *i*, respectively, and let **J**′*_i_*_−1_ = [*I*_1_ *I*_2_ … *I_i_*_−1_] be a vector of DMLPSIs values until Stage *i* − 1. We need to obtain the DMLPSI vector of coefficients at Stage *i* such that the covariance between *I_i_* and **J** *_i_*_−1_ will be null [i.e., cov(*I_i_*, **J** *_i_*_–1_) = **0**]. By this restriction, *I_i_* and *I_i_*_−1_ are not correlated; hence the name “decorrelated multi‐stage linear phenotypic selection index.” Xu and Muir (1992) showed that cov(*I_i_*, **J** *_i_*_–1_) = **B**′*_i_*_−1_ **Q**_(_ *_i_*_−1)_ *_i_* **b** *_i_*, where, $B_{i-1}$ is an upper triangular matrix (Cerón‐Rojas et al. 2019a) where each column contain the vector of coefficients of the DMLPSI, $\;b_{i-1}^{\prime }=\left[\begin{array}{cc} b_{(i-1)1} & b_{(i-1)2} \end{array}\;\begin{array}{cc} \text{…} & b_{\left(i-1\right)n_{i-1}} \end{array}\right]\;$ is the DMLPSI vector of coefficients at Stage *i* − 1, **Q**_(_ *_i_*_−1)_ *_i_* is as defined above, and **b** *_i_* is the DMLPSI vector of coefficients at Stage *i*.

Let **S** *_i_*_(_ *_i_*_−1)_ = **Q** *_i_*_(_ *_i_*_−1)_ **B** *_i_*_−1_ and **S**′*_i_*_(_ *_i_*_−1)_ = **S**_(_ *_i_*_−1)_ *_i_* = **B**′*_i_*_−1_ **Q**_(_ *_i_*_−1)_ *_i_* be the transpose of matrix **S** *_i_*_(_ *_i_*_−1)_, and assume that matrices **Q** *_ii_*, **Q** *_i_*_(_ *_i_*_−1)_, and **Λ** *_i_* and vector **w** are known. To minimize the mean squared difference between *H* = **w**′**g** and *I_i_* = **b**′*_i_* **ω** *_i_* (i.e., *E*[(*H* − *I_i_*)^2^]), under the restriction **S**_(_ *_i_*_−1)_ *_i_* **b** *_i_* = **B**′*_i_*_−1_ **Q**_(_ *_i_*_−1)_ *_i_* **b** *_i_* = **0**, it is necessary to minimize the function

$$f\left(b_{i},\xi \right)={b^{\prime }}_{i}Q_{ii}b_{i}-2w^{\prime }\Lambda_{i}b_{i}+2\xi^{\prime }S_{(i-1)i}b_{i}$$

with respect to vectors **b** *_i_* and **ξ**′ = [ξ_1_ ξ_2_ … ξ*_i_*_−1_], where **ξ** is a vector of Lagrange multipliers. In matrix notation, the derivative results of **b** *_i_* and **ξ** are

$$\left[\begin{array}{c} b_{i}\\ \xi \end{array}\right]={\left[\begin{array}{cc} Q_{ii} & S_{i(i-1)}\\ S_{(i-1)i} & 0 \end{array}\right]}^{-1}\left[\begin{array}{c} \Lambda_{i}w\\ 0 \end{array}\right]$$

from where the DMLPSI vector of coefficients that minimizes *E*[(*H* − *I_i_*)^2^] at Stage *i* can be written as

(16) $$b_{i}=K_{i}\beta_{i}$$

where **K** *_i_* = [**I** *_i_* − **Φ** *_i_*], **Φ** *_i_* = **Q** *_ii_* ^−1^ **S** *_i_*_(_ *_i_*_−1)_[**S**_(_ *_i_*_−1)_ *_i_* **Q** *_ii_* ^−1^ **S** *_i_*_(_ *_i_*_−1)_]^−1^ **S**_(_ *_i_*_−1)_ *_i_*, **β** *_i_* = **Q** *_ii_* ^−1^ **Λ** *_i_* **w** (Equation 14), **Q** *_ii_* ^−1^ is the inverse of matrix **Q** *_ii_*, and **I** *_i_* is an identity matrix of the same size as **Q** *_ii_*. When the restriction cov(*I_i_*,**J** *_i_*_−1_) = **0** is not imposed, **β** *_i_* = **b** *_i_*, and at Stage 1 (*i *= 1), **S** *_i_*_(_ *_i_*_−1)_ = **S**_1(0)_ = **Q**_1(0)_ **B**_0_ = 0, **Φ**_1_ = **Q**_11_^−1^ **S**_1(0)_(**S**_(0)1_ **Q**_11_^−1^ **S**_1(0)_)^−1^ **S**_1(0)_ = **0**, and **K** *_i_* = **K**_1_ = [**I** − **0**] = **I**, where **I** is an identity matrix of the same size as **Φ**_1_. Thus, **b**_1_ = **K**_1_ **β**_1_ = **Q**_11_^−1^ **Λ**_1_ **w** = **β**_1_, where **Q**_11_^−1^ is the inverse of matrix **Q**_11_^−1^, **Λ**_1_ = **C**_1_, and cov(**ω**_1_,**g**) = **C**_1_. The method to obtain Equation 16 is valid for all selection indices associated with DMLPSI.

The DMLPSI expected genetic gain per trait, selection response, and correlation at Stage *i* are similar to those of Equation 15 changing **β** *_i_* for **b** *_i_* = **K** *_i_* **β** *_i_*. The only difference between the DMLPSI and OMLPSI is the way the selection intensities are obtained. Xu and Muir (1992) described a general method to obtain the DMLPSI selection intensity, whereas Cerón‐Rojas et al. (2019a, 2019b) described a method to obtain the OMLPSI selection intensity in the two‐stage context. Methods to obtain the DMLPSI and OMLPSI selection intensities are valid for both the unconstrained and constrained multi‐stage indices.

#### 4.18.3 OMLPSI selection intensity

As we have seen in Equation 6, the selection intensity (*k*), in the single‐stage context, is related to the height of the ordinate of the normal curve *z*(τ*) and the proportion selected (*p*) as k = [*z*(τ*)]/*p*. Kempthorne and Nordskog (1959) have indicated that

$$p=\int_{\tau^{*}}^{\infty }\frac{1}{\sqrt{2\pi }}\exp\left\{-\frac{1}{2}\tau^{2}\right\}d\tau$$

from where it is evident that *k* = [*z*(τ*)]/*p* is a left truncated normal distribution (Arismendi, 2013; Wilhelm & Manjunath, 2010). A truncated distribution is a conditional distribution resulting when the domain of the parent distribution is restricted to a smaller region (Hattaway, 2010). The equation *k* = [*z*(τ*)]/*p* is also called the “hazard function,” the “hazard rate,” or the “inverse Mill's ratio” of the normal distribution (Rausand & Hϕyland, 2004).

Young (1964) showed that the intensity of selection for the two‐stage case could be obtained in a similar manner as *k* = [*z*(τ*)]/*p* (Equation 6). Thus, suppose that indices *I*_1_ and *I*_2_ have a joint normal distribution and can be transformed into the standardized random normal variables *T*_1_ = (*I*_1_ − *m*_1_)/σ_1_ and *T*_2_ = (*I*_2_ − *m*_2_)/σ with mean zero and variance 1, where *m*_1_ and *m*_2_ are the means, and σ_1_ and σ_2_ are the standard deviations of *I*_1_ and *I*_2_, respectively. In this case, the method of selection is to retain animals or plants with *T*_1_ ≥ *c*_1_ at Stage 1 and *T*_1_ + *T*_2_ ≥ *c*_2_ at Stage 2, where *c*_1_ and *c*_2_ – *T*_1_ are truncation points for *I*_1_ and *I*_2_, respectively. Thus, according to Young (1964), the selected population has the bivariate left truncated normal distribution with probability density function given by

$$h_{T_{1},T_{2}}\left(\tau_{1},\tau_{2}\right)=\frac{f_{T_{1},T_{2}}\left(\tau_{1},\tau_{2}\right)}{p}$$

where

$$\begin{array}{c} f_{T_{1},T_{2}}\left(\tau_{1},\tau_{2}\right)=\frac{1}{2\pi \sqrt{1-\rho_{12}^{2}}}\\ \;\times \;\exp\left\{-\frac{1}{2(1-\rho_{12}^{2})}\left[\tau_{1}^{2}+\tau_{2}^{2}-2\rho_{12}^{2}\tau_{1}^{2}\tau_{2}^{2}\right]\right\} \end{array}$$

and ρ_12_ is the correlation between *T*_1_ and *T*_2_. The total proportion selected (*p* = *q*_1_ *q*_2_) can now be written as

$$p=q_{1}q_{2}=\int_{c_{1}}^{\infty }\int_{c_{2}-\tau_{1}}^{\infty }f_{T_{1},T_{2}}\left(\tau_{1},\tau_{2}\right)d\tau_{2}d\tau_{1}$$

where *c*_1_ and *c*_2_ – τ_1_ are truncation points for *I*_1_ and *I*_2_, respectively. It is evident that when the number of stages increases, the number of variables in $f_{T_{1},T_{2}}\left(\tau_{1},\tau_{2}\right)$, and the number of integrals in *p* also increases.

By the foregoing results, in the multi‐stage selection context it is usual to fix a proportion (*p*) before selection is carried out and then, based on the fixed *p* value, to determine the proportion *q_i_* (*i* = 1, 2, …, *S*) for each stage under the restriction

$$p=\prod_{i=1}^{S}q_{i}$$

where *S* is the number of stages. In a two‐stage selection context, *p* = *q*_1_ *q*_2_. It is assumed that *p* is known but *q_i_* is unknown. One way of solving the equation *p* = *q*_1_ *q*_2_ is by trial and error, depending on the importance of each trait (Hazel & Lush, 1942) or each index at each stage. Cerón‐Rojas et al. (2019a, 2019b) have given a general method to estimate the OMLPSI selection intensities in the two‐stage context, whereas Xu and Muir (1992) have provided a general method to obtain the selection intensities for the DMLPSI. The methods to obtain the selection intensities described above are valid for any multi‐stage index. Until now, there has been no general procedure to obtain the OMLPSI selection intensity for any number of stages.

### 4.19 LGSI

Togashi et al. (2011) originally proposed the LGSI, which is a linear combination of GEBVs; however, Ceron‐Rojas et al. (2015) and Cerón‐Rojas and Crossa (2019) developed the complete unconstrained and contrained LGSI theory. These authors designed the LGSI for selecting genotypes in inbred populations; that is, LGSI exploits the linkage disequilibrium between markers and quantitative trait loci (QTL) produced when inbred lines are crossed to select parents for the next selection cycle. This index uses all available marker information in the prediction because it obtains the GEBV by multiplying the genomic best linear unbiased predictor of all estimated marker effects in the training population by the marker code values of the testing population (VanRaden, 2008).

#### 4.19.1 The genomic breeding values

Let **u** *_j_* be an *m* × 1 vector of QTL additive effects associated with markers that affect the *j*th trait, and let **L** be a matrix *n* × *m* (*n *= number of individual and *m* = number of markers in the population) of coded marker values (e.g., 2 – 2*q*, 1 – 2*q*, and –2*q* for the marker genotypes *AA*, *Aa*, and *aa*, respectively); then

(17) $$\alpha_{j}=Lu_{j}$$

is the vector of individual genomic breeding values associated with the *j*th characteristic (*j* = 1, 2, …, *t*; *t* = number of traits) of the candidates for selection. We assumed that **α** *_j_* has multivariate normal distribution with mean **0** and covariance matrix **G**σ_α_^2^, where σ_α_^2^ is the additive genomic variance of **α** *_j_*; **G** = **LL**′|κ is the matrix of additive genomic relationship, $\kappa =\sum_{l=1}^{m}2q_{l}\left(1-q_{l}\right)$ in an F_2_ population; *q_l_* is the frequency of allele *A*; and 1 − *q_l_* is the frequency of allele *a* for the *l*th marker (l = 1, 2, …, *m*).

#### 4.19.2 The net genetic merit and its relationship with the genomic breeding values

The net genetic merit is related to the vector of genomic breeding values (Equation 17) as

(18) $$H=\beta^{\prime }\alpha +e$$

where **β**′ = [β_1_ β_2_ … β*_t_*] is the vector of regression coefficients, **α**′ = [**α**_1_ **α**_2_ … **α** *_t_*], and **e** is the residual with normal distribution, null expectation, and variance σ*_e_* ^2^. It is possible to show that the covariance between **β**′**α** and **e** equals zero.

#### 4.19.3 The LGSI

The BLP of *H* is the conditional expectation of *H* given **α**, that is

(19) $$E\left(H|\alpha \right)=w^{\prime }\Gamma \Gamma^{-1}\alpha =w^{\prime }\alpha$$

where **w**′**Γ** = cov(*H*, **α**)′ is the covariance between *H* and α, **Γ** ^−1^ is the inverse matrix of the genomic covariance matrix **Γ** = var(**α**) = cov(**α**,**g**), **w** is as defined in Equation 2, and *I_G_* = **w**′**α** is the LGSI.

#### 4.19.4 The estimator of the LGSI

Let ${\hat{u}}_{j}$ be a predictor of the **u** *_j_* vector of QTL effects associated with the markers that affect the *j*th trait (Cerón‐Rojas & Crossa, 2019; Ceron‐Rojas et al., 2015); then the expectation of **u** *_j_* given ${\hat{u}}_{j}$ is $E\left(u_{j}|{\hat{u}}_{j}\right)={\hat{u}}_{j}$. This implies that the expectation of *H*, given the estimator of the LGSI,

$${\hat{I}}_{G}=w_{1}{\hat{\alpha }}_{1}+w_{2}{\hat{\alpha }}_{2}+\cdot +w_{t}{\hat{\alpha }}_{t}$$

is equal to

$$E\left(H|{\hat{I}}_{G}\right)={\hat{I}}_{G}$$

where ${\hat{\alpha }}_{j}=L{\hat{u}}_{j}$ is the vector of GEBVs associated with trait *j*th.

#### 4.19.5 Selection response and expected genetic gain per trait

By Equations 8–10, the LGSI selection response and expected genetic gain per trait are, respectively,

(20) $$R_{G}=k\sqrt{w'\Gamma w}\;\;\mathrm{and}\;\;E_{G}=k\frac{\Gamma w}{\sqrt{w'\Gamma w}}$$

We have previously defined all parameters of Equation 20. To simplify notation, in Equation 20 we omitted the intervals between selection cycles, which denote the time required for LGSI to complete one selection cycle (Ceron‐Rojas et al., 2015). Using the Cauchy–Schwarz inequality, Cerón‐Roja and Crossa (2020b) showed that in the ssymptotic context, when the number of markers and genotypes tend to be infinite, $R_{G}=k\sqrt{w'\Gamma w}$ is the the upper boundary for the unconstrained LPSI selection response. It is possible to show that, in the constext of the constrained LPSI selection response, there is a similar result (Cerón‐Roja & Crossa, 2019).

### 4.20 Relationships between the LPSI and LGSI selection response

In the asymptotic context, when the number of markers and genotypes tend to infinite, matrix **Γ** approaches matrix **C,** and, at the limit, we would expect that **Γ** = **C** (Cerón‐Rojas & Sahagún‐Castellanos, 2016). This means that matrix **P** = **C** + **R** approaches matrix **P** = **Γ** + **R**, from where **P** ^−1^ = (**Γ** + **R**)^−1^ = **Γ** ^−1^ − **Γ** ^−1^(**Γ** ^−1^ + R^−1^)^−1^ **Γ** ^−1^ is the inverse of matrix **P** (Searle et al., 2006), whereas **Γ** ^−1^ and **R** ^−1^ are the inverses of matrices **Γ** and **R**, respectively. In the asymptotic context, let **b** = **P** ^−1^ **Γw** be the LPSI vector of coefficients; then, the LPSI response can be written as

$$R=k\sqrt{b^{\prime }\mathrm{Pb}}=k\sqrt{w^{\prime }\Gamma P^{-1}\Gamma w}=k\sqrt{w^{\prime }\Gamma w-w^{\prime }{\left(\Gamma^{-1}+R^{-1}\right)}^{-1}w}$$

Assuming that **b**′**Pb** and **w**′**Γw** are positive semi‐definite (i.e., **b**′**Pb** ≥ 0 and **w**′**Γw** ≥ 0), then **w**′**Γw** ≥ **w**′(**Γ** ^−1^ + **R** ^−1^)^−1^ **w** ≥ 0, from where in the asymptotic context, *R_G_* ≥ *R*.

In the non‐asymptotic context, when the number of markers and genotypes is low, matrices **C** and **P** can be written as **C** = **Γ** + **Ξ** and **P** = (**Γ** + **Ξ**) + **R**, respectively, where **Ξ** = **C** − **Γ**. Thus, the inverse of matrix **P** is


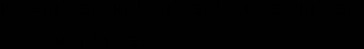

from where **b** = **P** ^−1^(**Γ** + **Ξ**)**w**, and the LPSI selection response (*R*) is

$$\begin{array}{ccc} R & = & k\sqrt{b\prime \mathrm{Pb}}=k\sqrt{w^{\prime }\left(\Gamma +\Xi \right)P^{-1}\left(\Gamma +\Xi \right)w}\\ & = & k\sqrt{w^{\prime }\Gamma w+w^{\prime }\Xi w-w^{\prime }{\left[{\left(\Gamma +\Xi \right)}^{-1}+R^{-1}\right]}^{-1}w} \end{array}$$

This last equation indicates that in the non‐asymptotic context, *R_G_* and *R* are related in three possible ways:

1. $R>R_{G}$ if $w^{\prime }\Xi w>w^{\prime }{\left[{\left(\Gamma +\Xi \right)}^{-1}+R^{-1}\right]}^{-1}w$
2. $R=R_{G}$ if $w^{\prime }\Xi w=w^{\prime }{\left[{\left(\Gamma +\Xi \right)}^{-1}+R^{-1}\right]}^{-1}w$
3. $R_{G}>R$ if $w^{\prime }\Xi w<w^{\prime }{\left[{\left(\Gamma +\Xi \right)}^{-1}+R^{-1}\right]}^{-1}w$

By Points 2 and 3, in the non‐asymptotic context, *R_G_* may be equal to or larger than *R* depending on the size of **w′** **Ξ** **w** and **w**′[(**Γ** + **Ξ**)^−1^ + **R** ^−1^]^−1^ **w**.

## 5 COMBINED LINEAR SELECTION INDICES

A combined LSI to predict *H* (Equation 2) is a linear combination of phenotypic values and marker scores (LMSI) or phenotypic values and GEBVs (called combined LGSI). Both indices combine information on markers linked to QTL and phenotypic values of the traits in the prediction of *H* because it is impossible to identify all QTL affecting the economically important traits (Dekkers, 2007; Dekkers & Settar, 2004; Lande & Thompson, 1990; Li, 1998). The LMSI assumes that favorable alleles and their average effects on phenotype are known; however, this assumption is only valid for traits conditioned by major genes and not for quantitative traits that are influenced by the environment. This is important because many QTL with small effects could interact with the environment and among themselves (Heffner et al., 2009; Hospital et al., 1997). Linear molecular selection index efficiency depends on various factors, such as number and density of markers associated with QTL, population size, trait heritability, additive genetic variances explained by markers, and the precision of the estimated effect of gene substitution (Dekkers & Dentine, 1991). Several authors (Gimelfarb & Lande, 1994, 1995; Zhang & Smith, 1992, 1993) have pointed out the effectiveness of the LMSI in inbred populations with large population sizes and traits with low heritability values when only one trait and its associated molecular score are considered. Moreau et al. (2000, 2007) found that the LMSI was more effective than LPSI only in early‐generation testing and that LMSI had the additional disadvantage of increased costs due to molecular marker evaluation.

In the genomic selection context, Dekkers (2007) developed the “combined LGSI” as a possible solution to LMSI problems. This index uses GEBVs (no marker scores) and phenotypic values jointly to predict *H* and is very similar to the LMSI. We describe the LMSI, the combined LGSI, and the combined optimum and decorrelated multi‐stage LGSI (OMLGSI and DMLGSI, respectively) developed by Cerón‐Rojas and Crossa (2020a).

### 5.1 The LMSI molecular score

Suppose that the QTL effects combine additively both within and between loci; then the *h* ^th^ unobservable genetic value *G_h_* can be written as

$$G_{h}=\sum_{k=1}^{N}\vartheta_{k}\upsilon_{k}$$

where ϑ*_k_* is the effect of the QTL *k* ^th^, υ*_k_* is the number of favorable alleles at the QTL *k* ^th^ (2, 1, or 0), and *N* is the number of QTL affecting the *j* ^th^ trait of interest. Because the QTL effect values are not observable, the *G_h_* values are also not observable; however, we can use a linear combination of the markers linked to the QTL (*s_h_*) that affect the *j^th^* trait to predict *G_h_* as

$$s_{h}=\sum_{k=1}^{M}b_{k}l_{k}$$

where *s_h_* is a predictor of *G_h_*, *b_k_* is the regression coefficient of the linear regression model, *l_k_* is the coded value of the *k* ^th^ markers (e.g., 1, 0, and −1 for marker genotypes *AA*, *Aa*, and *aa*, respectively), and *M* is the number of selected markers linked to the QTL that affect the *j* ^th^ trait. The above equation is called the “marker score” (Lande & Thompson, 1990). The number of selected markers is only a subset of potential markers linked to QTL in the population under selection; thus, the the variance of the *s_h_* values should be lower than or equal to the variance of the *G_h_* values.

### 5.2 The combined LSI

The combined LSI is

(22) $$I_{M}={\beta^{\prime }}_{y}y\;\;+\;\;{\beta^{\prime }}_{C}\varphi \;\;=\;\;\left[\begin{array}{cc} {\beta^{\prime }}_{y} & {\beta^{\prime }}_{C} \end{array}\right]\left[\begin{array}{c} y\\ \varphi \end{array}\right]\;\;=\;\;\beta^{\prime }t$$

where **φ** can be the vector of marker scores, **s**′ = [*s*_1_ *s*_2_ … *s_t_*], or the vector of genomic breeding values **α** (Equation 18). When **φ** = **s**, Equation 22 is the LMSI, whereas when **φ** = **α**, Equation 22 is the combined LGSI. Vector **y** was defined earlier, whereas **β**′ = [**β** *_y_* ′ **β**′*_C_*], and **t**′ = [**y**′ **φ**′].

### 5.3 Vector of coefficients, maximized selection response, and correlation

Let var(**s**) = **S** be the covariance matrix of marker score and **Γ** as defined in Equation 19; then, according to the LPSI theory, the vector of coefficients that maximizes the combined LSI selection response, expected genetic gain per trait, and correlation is

(23) $$\beta =O^{-1}\Pi \nu$$

where when $O=\mathrm{var}\left[\begin{array}{c} y\\ s \end{array}\right]=\left[\begin{array}{cc} P & S\\ S & S \end{array}\right]$ and $\Pi =\mathrm{var}\left[\begin{array}{c} g\\ s \end{array}\right]=\left[\begin{array}{cc} C & S\\ S & S \end{array}\right]$, Equation 23 is the LMSI vector of coefficients, whereas when $O=\mathrm{var}\left[\begin{array}{c} y\\ z \end{array}\right]$ and $\Pi =\mathrm{var}\left[\begin{array}{c} g\\ z \end{array}\right]$, Equation 23 is the combined LGSI vector of coefficients. Vector **v**′ = [**w**′ **0**′] is the vector of economic weights, where **0** is a null vector of size *t* × 1. The other parameters were previously defined.

### 5.4 The maximized combined LSI selection response

By Equation 23, the maximized LMSI and combined LGSI selection response of both indices is

(24) $$R_{C}=k\sqrt{\beta^{\prime }O\beta }$$

where *k* is the selection intensity. When **S** and **Γ** are null matrices, **β** *_y_* = **b** = **P** ^−1^ **Cw** (the LPSI vector of coefficients), whereas *R_C_* = *R* (the LPSI selection response). In addition, when the number of markers and genotypes increases, matrix **Γ** tends to matrix **C** and at the limit **Γ** = **C**, from where **β**_y_ = **0**, **β** *_C_* = **w**, and Equation 24 is equal to $R_{G}=k\sqrt{w^{\prime }\Gamma w}$ (Equation 20). That is, the weights and selection response of the LGSI and the combined LGSI will be equal.

### 5.5 The combined LGSI expected genetic gain per trait and correlation

The maximized combined LGSI expected genetic gain per trait (**E** *_C_*) and correlation ($\rho_{HI_{C}}$) are, respectively,

(25) $$E_{C}=k\frac{\Pi \beta }{\sqrt{\beta^{\prime }O\beta }}\;\mathrm{and}\;\rho_{HI_{C}}=\frac{\sqrt{\beta^{\prime }O\beta }}{\sqrt{w^{\prime }Cw}}$$

where *k* is the selection intensity. Note that the denominator of $\rho_{HI_{C}}$ (Equation 25) and the denominator of the maximized LPSI correlation (Equation 11) are very similar; however, the numerator of both equations is different. We would expect that $\sqrt{\beta^{\prime }O\beta }\geq \sqrt{b^{\prime }\mathrm{Pb}}$, and then $\rho_{HI_{C}}\geq \rho_{HI}$.

### 5.6 Asymptotic relationship between combined LGSI and LGSI parameters

The asymptotic relationship between **E** *_C_* (Equation 25) and the LGSI expected genetic gain per trait $E_{G}$ (Equation 20) is as follows. When Γ=C, $\Pi =\left[\begin{array}{cc} \Gamma & \Gamma \\ \Gamma & \Gamma \end{array}\right]$ and $\beta^{\prime }=\left[\begin{array}{cc} 0^{\prime } & w^{\prime } \end{array}\right]$, from where

(26) $$E_{C}=k\frac{2\Gamma w}{\sqrt{w^{\prime }\Gamma w}}=2E_{G}$$

This means that in the asymptotic context, the CLGSI expected genetic gain per trait is twice the LGSI expected genetic gain per trait. Of course, 2 is only a proportionality constant; thus, in reality, $E_{C}=E_{G}$. Similarly, when **Γ** = **C** and **β**′ = [**0**′ **w**′], **β**′**O** **β** = **w**′**Γw** and **w**′**Cw** = **w**′**Γw** in $\rho_{HI_{C}}$(Equation 25); thus, the maximum correlation between *H* and *I_C_* in the asymptotic context will be equal to 1.0, as we would expect.

### 5.7 Combined multi‐stage LGSIs

Based on the LMSI, Xie and Xu (1998) developed a decorrelated multi‐stage LMSI. They assumed that their index would increase the efficiency of molecular assisted selection in a two‐stage context by first (Stage 1) selecting immature seedlings (embryos) based on molecular score information only, and later, at Stage 2, by selecting individual traits based on molecular score and phenotypic value information jointly. The Xie and Xu (1998) index has the same LMSI problems as previously indicated. For this reason, Cerón‐Rojas and Crossa (2020a) adapted the Dekkers (2007) index to the multi‐stage context using the OMLPSI and DMLPSI approaches. Both these indices use GEBVs and phenotypic information jointly to predict the net genetic merit; then they are free of the problems of the Xie and Xu (1998) index. In the multi‐stage context, the Cerón‐Rojas and Crossa (2020a) indices are as follows: at Stage 1, we can select immature (seedlings, embryos) based on GEBVs information only and, in Stage 2, we select individual traits based on GEBVs and phenotypic values jointly. This is the Xie and Xu (1998) idea but in the genomic selection context.

The approach based on the OMLPSI optimum index will be called “optimum combined multi‐stage LGSI” (OCMLGSI), whereas the index based on the DMLPSI will be called “decorrelated combined multi‐stage LGSI” (DCMLGSI) because, at stage two, both indices use GEBVs and phenotypic information jointly to predict the net genetic merit.

### 5.8 The OCMLGSI and DCMLGSI parameters at Stage 1

The LGSI and LPSI are part of the OCMLGSI and DCMLGSI. At stage‐one the estimator of the LGSI (${\hat{I}}_{G}=w_{1}{\hat{\alpha }}_{1}+w_{2}{\hat{\alpha }}_{2}+\cdot +w_{t}{\hat{\alpha }}_{t}$, where ${\hat{\alpha }}_{j}=L{\hat{u}}_{j}$ is the vector of GEBV of trait *j* ^th^) is used to predict *H* because at this stage, both indices select immature seedlings (or embryos) based only on GEBV information. The OCMLGSI and DCMLGSI maximized selection responses are, respectively,

(27) $$R_{O_{1}}=k_{O_{1}}\sqrt{w^{\prime }\Gamma w}\;\mathrm{and}\;R_{D_{1}}=k_{D_{1}}\sqrt{w^{\prime }\Gamma w}$$

where $k_{O_{1}}$ and $k_{D_{1}}$ (the selection intensities for each index) are the only difference between $R_{O_{1}}$ and $R_{D_{1}}$. The maximized correlation between *H* = **w**′**g** and *I_G_* = **w**′α is $\rho_{{}_{\mathrm{HI}}}=\frac{\sqrt{w^{\prime }\Gamma w}}{\sqrt{w^{\prime }Cw}}$, where $\sqrt{w^{\prime }\Gamma w}$ is the standard deviation of the variance of *I_G_* = **w**′**α**, $\sigma_{H}=\sqrt{w^{\prime }Cw}$ is the standard deviation of *H* = **w**′**g**, and **C** is the covariance matrix of **g**.

### 5.9 Covariance matrices for OCMLGSI and DCMLGSI

The main difference between the multi‐stage LPSIs described earlier (OMLPSI and DMLPSI) and OCMLGSI and DCMLGSI is that, in addition to **y**′ = [*Y*_1_ *Y*_2_ … *Y_t_*], the latter two indices incorporate **α** (Equation 18) in the prediction of *H*. Let **η**′ = [**α**′ **y**′]; then vector **η** can be partitioned into *S* subvectors as **η**′ = [**α**′ **y**′] = [**ω**′_1_ **ω**′_2_ … **ω**′*_S_*], where vector **y** has been partitioned into *S* − 1 subvector as **y**′ = [**ω**′_2_ **ω**′_3_ … **ω**′*_S_*]. This means that at Stage 1, **ω**_1_ = **α** (Equation 18), and *I_i_* = **β**′*_i_* **ω** *_i_* is the index at Stage *i* (*i* = 2, 3, …, *S*), where $\beta_{i}^{\prime }=\left[\begin{array}{cc} \beta_{i1} & \beta_{i2} \end{array}\;\begin{array}{cc} \text{…} & \beta_{it_{i}} \end{array}\right]\;$.

Let **g**′ = [G_1_ G_2_ … G*_t_*] be a vector of true unobservable random breeding values for *t* traits, and let **α**, **y**, and **ω**_1_ be as defined earlier. The covariance between **α** *_j_* and G*_j_* (*j* = 1, 2, …, *t*) is equal to the variance of **α** *_j_* [i.e., cov(**α** *_j_*, *G_j_*) = σ_α_^2^] (Dekkers, 2007); the covariance matrix of **α** is var(**α**) = **Γ**, and the covariance matrices between **ω** *_i_* (*i* = 1, 2, …, *S*) and **g** for *S* stages is

$$\left[\begin{array}{c} \mathrm{cov}\left(\omega_{1},g\right)\\ \mathrm{cov}\left(\omega_{2},g\right)\\ \begin{array}{c} \vdots \\ \mathrm{cov}\left(\omega_{S},g\right) \end{array} \end{array}\right]=\left[\begin{array}{c} \Gamma \\ C_{2}\\ \begin{array}{c} \vdots \\ C_{S} \end{array} \end{array}\right]\;=\Omega_{\Gamma }$$

where at Stage 1, **ω**_1_ = **α**, cov(**ω**_1_, **g**) = **Γ** is the genomic covariance matrix, and from Stage 2 to *S*, cov(**ω** *_i_*, **g**) = **C** *_i_* is the *i* ^th^ genotypic covariance submatrix of **Ω**_Γ_. Note that while the size of matrix **Γ** is *t* × *t*, the size of matrix **C** *_i_* will be *n_i_* × *t*, where *n_i_* is the size of vector **ω** *_i_*. For example, if **g**′ = [*g*_1_ *g*_2_ *g*_3_ *g*_4_], **ω**′_1_ = [**α**_1_ **α**_2_ **α**_3_ **α**_4_], and **ω**′_1_ = [*Y*_1_ *Y*_2_], then **Γ** will be of size 4×4, and **C**_2_ will be of size 2×4, where 2 is the number of rows and 4 is the number of columns in matrix **C**_2_. By the foregoing results, the covariance matrix of **η**′ = [**α**′ **y**′] = [ω′_1_ ω′_2_ … ω′*_S_*] is

$$\sum \;=\left[\begin{array}{c} \begin{array}{cc} \Gamma & \Gamma_{12}\\ \Gamma_{21} & P_{22}\; \end{array}\begin{array}{cc} \cdots & \Gamma_{1S}\\ \cdots & P_{2S} \end{array}\\ \begin{array}{cc} \vdots & \vdots \\ \Gamma_{S1} & P_{S2}\; \end{array}\begin{array}{cc} \ddots & \vdots \\ \cdots & P_{\mathrm{SS}} \end{array} \end{array}\right]$$

where all the gamma matrices are genomics matrices, while the others are phenotypic covariance matrices. Matrices **Q** *_ii_* and **Λ** *_i_* are constructed as

$$Q_{ii}=\left[\begin{array}{c} \begin{array}{cc} \Gamma & \Gamma_{12}\\ \Gamma_{21} & P_{22}\; \end{array}\begin{array}{cc} \text{…} & \Gamma_{1i}\\ \text{…} & P_{2i} \end{array}\\ \begin{array}{cc} \vdots & \vdots \\ \Gamma_{i1} & P_{i2}\; \end{array}\begin{array}{cc} \ddots & \vdots \\ \text{…} & P_{ii} \end{array} \end{array}\right]\;\mathrm{and}\;\Lambda_{i}=\left[\begin{array}{c} \Gamma \\ C_{2}\\ \vdots \\ C_{i} \end{array}\right]$$

which are submatrices of **∑** and **Ω** **_Γ_**, respectively, until Stage *i*.

### 5.10 The OCMLGSI and DCMLGSI at Stage 2

At Stage 2, OCMLGSI and DCMLGSI is

(28) $$I_{C}={\beta^{\prime }}_{G}\alpha +{\beta^{\prime }}_{y}y=\left[\begin{array}{cc} {\beta^{\prime }}_{G} & {\beta^{\prime }}_{y} \end{array}\right]\left[\begin{array}{c} \alpha \\ y \end{array}\right]={\beta^{\prime }}_{2}t^{*}$$

where **y**′ = [**ω**′_2_ **ω**′_3_ … **ω**′*_S_*] and α is as defined earlier; **β** *_G_* and **β** **_y_** are vectors of coefficients of genomic and phenotypic weight values, respectively, **β**′_2_ = [**β**′*_G_* **β**′**_y_**], and **t**′* = [**α**′ **y**′]. The only difference between OCMLGSI and DCMLGSI is the way vector **β**′_2_ = [**β**′*_G_* **β**′**_y_**] is obtained.

### 5.11 The OCMLGSI parameters at Stage 2

The OCMLGSI vector of coefficients (**β**_2_) that maximizes the response, correlation, and expected genetic gain per trait (Equation 25) at Stage 2 is

(29) $$\beta_{2}=Q_{22}^{-1}\Lambda_{2}\nu^{*}$$

where $Q_{22}^{-1}$ is the inverse of matrix $Q_{22}=\left[\begin{array}{cc} \Gamma & \Gamma \\ \Gamma & P_{22} \end{array}\right]$, where ${\omega^{\prime }}_{2}^{*}=\left[\begin{array}{cc} {\omega^{\prime }}_{1} & {\omega^{\prime }}_{2} \end{array}\right]$ and $P_{22}=\mathrm{var}\left(\omega_{2}\right)$; $\Lambda_{2}=\left[\begin{array}{c} \Gamma \\ C_{2} \end{array}\right]$, $C_{2}=\mathrm{cov}\left(g,\omega_{2}\right)$, ${\nu^{\prime }}^{*}=\left[\begin{array}{cc} 0^{\prime } & w^{\prime } \end{array}\right]$ is a vector where **0** is a null vector of size *t* × 1, and **w** is the vector of economic weights. The maximized selection response, and the maximized correlation between *H* and OCMLGSI, respectively, are

(30) $$R_{O_{2}}=k_{O_{2}}\sqrt{{\beta^{\prime }}_{2}Q_{22}\beta_{2}}\;\mathrm{and}\;\rho_{HI_{C}}=\frac{\sqrt{{\beta^{\prime }}_{2}Q_{22}\beta_{2}}}{\sqrt{w^{\prime }Cw}}$$

where $k_{O_{2}}$ is the OCMLGSI intensity at stage two. The total selection response for Stages 1 and 2 is $R_{O_{t}}=R_{O_{1}}+R_{O_{2}}$.

### 5.12 The DCMLGSI parameters at Stage 2

The process to obtain the DCMLGSI vector of coefficients is similar to that described in subsection “The DMLPSI vector of coefficients at Stage *i*,” where we described how to obtain the DMLPSI vector of coefficients (Equation 16). Subsequently, in this subsection we will only provide the main results of the DCMLGSI for Stage 2. Additional details associated with DCMLGSI are in Cerón‐Rojas and Crossa (2020a).

The DCMLGSI vector of coefficients at Stage 2 is

(31) $$b_{2}=K_{2}\beta_{2}$$

where **K**_2_ = [**I**_2_ − **Φ**_2_], **Φ**_2_ = **Q**_22_^−1^ **S**_21_[**S**_12_ **Q**_22_^−1^ **S**_21_]^−1^ **S**_12_, and **β**_2_ = **Q**_22_^−1^ **Λ**_2_ **α** (Equation 29); and **I**_2_ is an identity matrix of the same size as matrix **Q**_22_, **S**_21_ = **Q**_21_ **B**_1_; and **S**′_21_ = **S**_12_ = **B**′_1_ **Q**_12_ is the matrix of constraints similar to the matrix defined in the DMLPSI context. Matrix **K**_2_ transforms the OCMLGSI vector of coefficients (**β**_2_) into Equation 31 and is the only difference with respect to Equation 29. The maximized DCMLGSI selection response and correlation for two stages can be written as in Equation 30 by changing **β**_2_ = **Q**_22_^−1^ **Λ**_2_ ν* by Equation 31 and the OCMLGSI selection intensity ($k_{O_{2}}$) by the DCMLGSI selection intensity ($k_{D_{2}}$). Finally, note that at Stage 2 **B**′_1_ = [**w**′ **0**′*_S_*_−1_], where **w** = **b**_1_ is the vector of economic weights and **0**′*_S_*_−1_ is a null vector of size *S* − 1. The size of matrix **Q**_12_ depend on the number of traits selected at Stage 2.

## 6 CONSTRAINED LINEAR SELECTION INDICES

By imposing null restrictions on Equations 10, Kempthorne and Nordskog (1959) developed the null restricted LPSI (denoted RLPSI) in the single‐stage context, which imposes restrictions equal to zero on the trait expected genetic gain. Later, Mallard (1972) generalized the RLPSI and developed an optimum constrained LPSI (CLPSI), which imposes restrictions different to zero and includes the RLPSI as a particular case. Both indices mainly affect the expected genetic gain per trait (Equation 10). Whereas in the single‐stage context the CLPSI vector of coefficients is a linear combination of the LPSI vector of coefficients (**β** = **P** ^−1^ **Cw**), in the multi‐stage context, the constrained optimum multi‐stage LPSI (COMLPSI) vector of coefficients is a linear combination of the OMLPSI vector of coefficients (Equation 14). In turn, the CDMLPSI vector of coefficients is also a linear combination of the OMLPSI vector of coefficients.

### 6.1 Single‐stage CLPSI

#### 6.1.1 CLPSI main objective and Mallard matrix

Assume that μ*_j_* is the population mean of the *j* ^th^ trait before selection. The main objective of the CLPSI is to change μ*_j_* to μ*_j_* + *d_j_*, where *d_j_* is a predetermined change in μ*_j_* imposed by the breeder on Equation 10. Let

$$N^{\prime }=\left[\begin{array}{ccccc} d_{r} & 0 & \text{…} & 0 & -d_{1}\\ 0 & d_{r} & \text{…} & 0 & -d_{2}\\ \vdots & \vdots & \ddots & \vdots & \vdots \\ 0 & 0 & \text{…} & d_{r} & -d_{r-1} \end{array}\right]\;$$

a Mallard (1972) matrix (*r* − 1) × *r* of predetermined proportional gains, where *d_j_* (*j *= 1, 2, …, *r*) is the *j* ^th^ element of vector **d**′ = [*d*_1_ *d*_2_ … *d_r_*], and let **U**′ be a matrix (*t* − *r*) × *t* (*t* = number of traits; *r* = number of restricted traits) of 1s and 0s, where 1 indicates that the traits are restricted, and 0 indicates that the traits are unrestricted. The size of matrix **U**′ depends on the number of restricted traits.

#### 6.1.2 Matrix **U**′

We can construct matrix **U**′ as follows. Suppose that we restrict one of the *t* traits; then, we can restrict the first of them as **U**′ = [1 0 0 … 0], the second one as **U**′ = [0 1 0 … 0], etc. In addition, we can restrict the first and second traits jointly as $U^{\prime }=\left[\begin{array}{ccc} 1 & 0 & 0\\ 0 & 1 & 0 \end{array}\begin{array}{c} \text{…}\\ \cdots \end{array}\begin{array}{c} 0\\ 0 \end{array}\right]\;$, the first and third traits as $U^{\prime }=\left[\begin{array}{ccc} 1 & 0 & 0\\ 0 & 0 & 1 \end{array}\begin{array}{c} \text{…}\\ \cdots \end{array}\begin{array}{c} 0\\ 0 \end{array}\right]\;$, the second and third traits as $U^{\prime }=\left[\begin{array}{ccc} 0 & 1 & 0\\ 0 & 0 & 1 \end{array}\begin{array}{c} \text{…}\\ \cdots \end{array}\begin{array}{c} 0\\ 0 \end{array}\right]\;$, etc. The procedure used to construct matrix **U**′ is valid for any number of restricted traits (Cerón‐Rojas & Crossa, 2018).

#### 6.1.3 The CLPSI vector of coefficients

Let **M**′ = **N**′**Ψ**′ be a Mallard (1972) matrix of restrictions, where **Ψ**′ = **U**′**C** is a matrix of null restrictions, and **N**′, **U**′, and **C** are as defined earlier. To obtain the CLPSI vector of coefficients we need to minimize the mean squared difference *E*[(*I* − *H*)^2^] under the restriction **M**′**β** = **0** assuming that **P**, **C**, **U**′, **N**′, and **w** are known; that is, we need to minimize the function

$$f\left(\beta ,v\right)=\beta^{\prime }P\beta +w^{\prime }Cw-2w^{\prime }C\beta +2\xi^{\prime }M^{\prime }\beta$$

with respect to vectors **β** and **ξ**′ = [ξ_1_ ξ_2_ … ξ*_r_*_−1_], where **ξ** is a vector of Lagrange multipliers. The derivative results from **β** and **ξ** are

$$\left[\begin{array}{cc} P & M\\ M^{\prime } & 0 \end{array}\right]\left[\begin{array}{c} \beta \\ \xi \end{array}\right]=\left[\begin{array}{c} \mathrm{Cw}\\ 0 \end{array}\right]$$

from where the CLPSI vector of coefficients that minimizes *E*[(*I* − *H*)^2^] under the restriction **M**′**β** = **0** is

(32) $$\beta_{C}=K\beta$$

where **K** = [**I** *_t_* − **D**], **D** = **P** ^−1^ **M**(**M**′**P** ^−1^ **M**)**M**′, and **I** *_t_* is an identity matrix of size *t* × *t*. It is possible to show that when **N** = **U**, the restriction **M**′**β** = **0** changes to **Ψ**′**β** = **0**, from where Equation 32 changes to the RLPSI vector of coefficients, that is,

(33) $$b_{R}=K_{R}\beta$$

where **K** *_R_* = [**I** − **D**], **D** = **P** ^−1^ **Ψ**(**Ψ**′**P** ^−1^ **Ψ**)^−1^ **Ψ**′, **P** ^−1^ is the inverse of matrix **P**, and **I** is an identity matrix *t* × *t*. If **N** = **U** and **U** is a null matrix, then **b** *_R_* = **β** (the LPSI vector of coefficients). This means that the CLPSI is the most general index and it includes the RLPSI and LPSI as particular cases.

Using restriction **U**′**C**′**β** = θ*d, Tallis (1985) showed that Equation 32 can be written as

(34) $$b_{T}=b_{R}+\theta^{*}\delta^{*}$$

where **b** *_R_* = **K** *_R_* **β** (Equation 34), δ* = **P** ^−1^ **Ψ**(**Ψ**′**P** ^−1^ **Ψ**)^−1^ **d**, **d**′ = [*d*_1_ *d*_2_ … *d_r_*], and θ* = [**β**′**Ψ**(**Ψ**′**P** ^−1^ **Ψ**)^−1^ **d**]/[**d**′(**Ψ**′**P** ^−1^ **Ψ**)^−1^ **d**]. When θ* = 0, **b** *_T_* = **b** *_R_*, and if θ* = 0 and **U**′ is a null matrix, **b** *_T_* = **β** (Equation 14). In addition, when θ* = 1.0, Equation 34 is equal to **b** *_T_* = **b** *_R_* + δ*, which is not an optimum CLPSI (Mallard, 1972; Tallis, 1962). Itoh and Yamada (1987) have shown that Equation 34 can be written as Equation 32, and vice versa. Thus, Equations 32 and 34 express the same result in a different mathematical way.

There are some problems associated with θ*. For example, the θ* values should be higher than zero and θ* ≠ 1.0 because when the value of θ* is negative, the CLPSI moves the population trait means in the opposite direction to the desired direction (Itoh & Yamada, 1987). Problems associated with the RLPSI and CLPSI are (a) due to the constraints, the correlation between RLPSI and CLPSI with the net genetic merit decreases as the number of constraints increases and (b) the CLPSI may change the means in the opposite direction to the desired direction due to the opposite sign values between **w** and **d**.

According to Itoh and Yamada (1987), one possible solution to the above problems could be to use the desired gains LPSI theory (Brascamp, 1984; Itoh & Yamada, 1986) to make a selection. The most important aspect of the desired LPSI gains is that it does not require economic weights, which means that the covariance between *I* and *H* [Cov(*H*,*I*) = **w**′**C** **β**] is not defined. In addition, this index maximizes neither the correlation between *I* and *H* (ρ*_IH_*) nor the selection response (Equation 9). Another problem with this index is that it is not a predictor of *H* = **w**′**g**. Thus, we think that the CLPSI is a better option to select than the desired gains LPSI.

### 6.2 The maximized CLPSI expected genetic gain per trait, correlation, and response

By changing vector **β** = **P** ^−1^ **Cw** in Equations 32, 33, or 34 in Equations 10–12, it is possible to obtain the maximized CLPSI expected genetic gain per trait, correlation, and selection response. That is, the LPSI and CLPSI parameters are equivalent, and the only difference is how the vector of coefficients is obtained.

### 6.3 COMLPSI

According to the single‐stage CLPSI (Equation 32) and OMLPSI theory described above, to obtain the COMLPSI vector of coefficients at Stage *i* we need to minimize the mean squared difference between the net genetic merit *H* = **w**′**g** and the index *I_i_* = **β** *_i_* ′ **ω** *_i_*, i.e., *E*[(*H* − *I_i_*)^2^], under the restrictions **M** *_i_* ′ **β** *_i_* = **0**, where **M** *_i_* ′ = **N**′**U**′**Λ** *_i_* ′ and **Λ** *_i_* ′ is as defined earlier. Supposing that matrices **Q** *_ii_*, **U,** and **Λ** *_i_* ′ and vectors **d** and **w** are known, then at Stage *i* it is necessary to minimize the function

$$f_{O}\left(\beta_{i},v\right)={\beta^{\prime }}_{i}Q_{ii}\beta_{i}-2w{\Lambda^{\prime }}_{i}\beta_{i}+2\xi^{\prime }{M^{\prime }}_{i}\beta_{i}$$

concerning the vector of coefficients **β** *_i_* and the vector of Lagrange multipliers **ξ**′ = [ξ_1_ ξ_2_ … ξ*_r_*_−1_]. The COMLPSI vector of coefficients at Stage *i* is

(35) $$\beta_{C_{i}}=K_{O_{i}}\beta_{i}$$

where **β** *_i_* is as defined in Equation 14, $K_{O_{i}}=\left[I_{i}-F_{O_{i}}\right]$, $F_{O_{i}}=Q_{ii}^{-1}M_{i}{\left({M^{\prime }}_{i}Q_{ii}^{-1}M_{i}\right)}^{-1}{M^{\prime }}_{i}$, and **I** *_i_* is an identity matrix of the same size as **Q** *_ii_*. When **N** = **U**, the vector of coefficients of Equation 35 is similar to Equation 33, and when **N** = **U** is a null matrix, Equation 35 is equal to Equation 14. Thus, the COMLPSI is more general than the OMLPSI and includes the OMLPSI and the multi‐stage null phenotypic restricted index as particular cases.

### 6.4 CDMLPSI

Xie and Xu (1997) extended the DMLPSI to a constrained DMLPSI (CDMLPSI); however, their approach is based on the single‐stage Tallis (1962) constrained index, which is not optimal. Based on the Mallard (1972) index (Equation 32), which is an optimal index, Cerón‐Rojas et al. (2019b) developed an optimal CDMLPSI, which we will describe in this subsection. The main difference between the CDMLPSI and the COMLPSI is that the COMLPSI imposes only one restriction when it solves the LPSI equations to obtain its vector of coefficients, whereas the CDMLPSI imposes the additional restriction that the covariance among the CDMLPSI values between stages be zero. As we shall see, the CDMLPSI vector of coefficients is a linear combination of the OMLPSI vector of coefficients (Equation 14).

Let *I_i_*_−1_ = **δ**′*_i_*_−1_ **ω** *_i_*_−1_ and *I_i_* = **δ**′*_i_* **ω** *_i_* be the CDMLPSI at Stages *i* − 1 and *i*, respectively, and let **J**′*_i_*_−1_ = [*I*_1_ *I*_2_ … *I_i_*_−1_] be a vector of CDMLPSIs values until Stage *i* − 1 so that the covariance between *I_i_* = **δ**′*_i_* **ω** *_i_* and **J**′*_i_*_−1_ is null. To obtain **δ** *_i_*, we need to minimize the mean squared difference between *H* = **w**′**g** and *I_i_* = **δ**′*_i_* **ω** *_i_* {*E*[(*H* − *I_i_*)^2^]} under the joint restrictions **M**′*_i_* **δ** *_i_* = **0** and cov(*I_i_*, **J** *_i_*_−1_) = **B**′_(_ *_i_*_−1)_ **Q**_(_ *_i_*_−1)_ *_i_* **δ** *_i_* = **0**, where **M**′*_i_* = **N**′**U** **′Λ′** *_i_*, and **Λ**′*_i_* is as defined earlier. In this case, **B** *_i_*_−1_ is an upper triangular matrix (Cerón‐Rojas et al., 2019a, 2019b) where each column contains the vectors of coefficients of the CDMLPSI until Stage *i* − 1.

In a manner similar to the DMLPSI, let **S** *_i_*_(_ *_i_*_−1)_ = **Q** *_i_*_(_ *_i_*_−1)_ **B**_(_ *_i_*_−1)_ and **S**′*_i_*_(_ *_i_*_−1)_ = **S**_(_ *_i_*_−1)_ *_i_*; then at Stage *i*, the CDMLPSI vector of coefficients that maximizes Equations 9 and 10 and the correlation is

(36) $$\delta_{i}=K_{D_{i}}\beta_{i}$$

where **β** *_i_* is as defined in Equation 14, $K_{D_{i}}=\left[I_{i}-F_{D_{i}}\right]$, $F_{D_{i}}=Q_{ii}^{-1}V_{i}{\left({V^{\prime }}_{i}Q_{ii}^{-1}V_{i}\right)}^{-1}{V^{\prime }}_{i}$, $V_{i}=\left[\begin{array}{cc} M_{i} & S_{i(i-1)} \end{array}\right]$, ${V_{i}}^{\prime }=\left[\begin{array}{c} M{"}_{i}\\ S_{(i-1)i} \end{array}\right]$, and **I** *_i_* is an identity matrix of the same size as **Q** *_ii_*. When matrix **S** *_i_*_(_ *_i_*_−1)_ is null, Equations 36 and 35 are the same. When **N** = **U**, Equation 36 imposes null restrictions, giving us a null restricted DMLPSI index similar to Equation 33.

According to Equations 35 and 36, matrices $K_{O_{i}}=\left[I_{i}-F_{O_{i}}\right]$ and $K_{D_{i}}=\left[I_{i}-F_{D_{i}}\right]$ transform the OMLPSI vector of coefficients (**β** *_i_*) into the COMLPSI and the CDMLPSI vectors of coefficients, respectively. In addition, the CDMLPSI expected genetic gain per trait, selection response, and the correlation at Stage *i* are the same as those of the OMLPSI when replacing **β** *_i_* with $\delta_{i}=K_{D_{i}}\beta_{i}$.

### 6.5 The single‐stage constrained LGSI

In a manner similar to the single‐stage CLPSI context (Equation 32), it can be shown (Cerón‐Rojas & Crossa, 2019) that the difference between the unconstrained LGSI described above and the constrained LGSI (CLGSI) is the projection matrix **K** *_G_* = **[I** *_t_* − **D** *_G_*], where **D** *_G_* = **UN**(**N**′**U**′**ΓUN**)^−1^ **N**′**U**′**Γ**. Matrices **N** and **U** were defined in Equation 32, whereas **I** *_t_* is an identity matrix *t* × *t*. Matrix **K** *_G_* is idempotent and projects the LGSI vector of coefficients (**w** = **β**) into a space that is smaller than the original space of **w**. Thus, while **w** is the vector of coefficients of the unconstrained LGSI,

(37) $$b_{G}=K_{G}w$$

is the CLGSI vector of coefficients. In the CLGSI context, the selection response and expected genetic gain per trait are the same as those described in Equation 20 when changing **w** for **b** *_G_* = **K** *_G_* **w**.

## 7 ESIM, RESIM, and PPG‐ESIM

In the current review, we describe ESIM, RESIM, and PPG‐ESIM, which were developed in the canonical correlation context, and do not use economic weights when predicting *H* = **w**′**g** and making selections. The other indices associated with ESIM (e.g., MESIM and GESIM) can be seen in Cerón‐Rojas and Crossa (2018).

### 7.1 ESIM

The fundamental difference between ESIM and the LPSI is the interpretation of the vector **w**. Whereas **w** is a vector of economic weights (known and fixed) in the LPSI context, in ESIM **w** is fixed but unknown and can be estimated in each selection cycle as a linear combination of the ESIM vector of coefficients. Moreover, in ESIM, the breeding values (**g**) and the phenotypic records (**y**) are two sets of variables that should have maximum correlation (λ*_j_*), from where the CCA theory is the base of ESIM. The ESIM index can be written as *I* = **b**′*_E_* **y**, where **b**′_E_ = [*b*_1_ *b*_2_ … *b*_t_] is the unknown index vector of coefficients, and *t* is the number of traits. In the CCA context, the vector of ESIM coefficients (**b**′*_E_*) is called the “canonical vector,” whereas λ*_j_* is called the “canonical correlation” (Wilms & Croux, 2016).

For the *j*th correlation (λ*_j_*), the CCA theory allows us to write ESIM and *H* = **w**′**g** as $I_{E}={b^{\prime }}_{E_{j}}y$ and $H_{E}={w^{\prime }}_{E_{j}}g$, respectively, where $w_{E_{j}}=C^{-1}Pb_{E_{j}}$, and $b_{E_{j}}$and λ*_j_* are obtained from

(38) $$\left(P^{-1}C-\lambda_{j}^{2}I\right)b_{E_{j}}=0$$

Note that $b_{E_{j}}$ is the *j*th vector (*j* = 1, 2, …, *t*) of the multi‐trait heritability matrix **P** ^−1^ **C** and maximizes the correlation ρ_HI_ = σ_HI_/σ_H_σ_I_ described in Equation 9 (Cerón‐Rojas & Crossa, 2018). Equation 38 is the ESIM fundamental results obtained by Cerón‐Rojas, Sahagún‐Castellanos, et al. (2008) and is the base for constructing RESIM, PPG‐ESIM, MESIM, and GESIM. To select with ESIM, breeders should use the first eigenvector ($b_{E_{1}}$) of matrix **P** ^−1^ **C** in $I_{E}={b^{\prime }}_{E_{1}}y$ and the first λ_1_ in the ESIM selection response.

### 7.2 Changing the signs and proportions of the ESIM vector of coefficients

Equation 38 can be written as $\left(P^{-1}C\right)Ib_{E_{j}}=\lambda_{j}^{2}Ib_{E_{j}}$, where **I** = **F** ^−1^ **F** is an identity matrix of size *t* × *t* (*t* = number of traits), and **F** = diag{*f*_1_ *f*_1_ … *f*_t_} is a diagonal matrix with values equal to any real number, except zero values. Thus, another way of writing Equation 38 is

$$\left[F\left(P^{-1}C\right)F^{-1}-\lambda_{j}^{2}I\right]{\beta_{E}}_{{}_{j}}=0$$

where $\beta_{E_{j}}=Fb_{E_{j}}$. Matrices **P** ^−1^ **C** and **F**(**P** ^−1^ **C**)**F** ^−1^ are similar, and both have the same eigenvalues but different eigenvectors (Harville, 2008). When the **F** values are only 1s, vector $b_{E_{j}}$ is not affected; when the **F** values are only −1s, vector $b_{E_{j}}$ will change its direction, and if the **F** values are different from 1 and −1, matrix **F** will change the proportional values of $b_{E_{j}}$. In practice, $b_{E_{j}}$ is first obtained from Equation 38 and then multiplied by matrix **F** to obtain $\beta_{E_{j}}=Fb_{E_{j}}$; that is, $\beta_{E_{j}}$ is a linear transformation of $b_{E_{j}}$. Matrix **F**(**P** ^−1^ **C**)**F** ^−1^ is called “similarity transformation,” and matrix **F** is called the “transforming matrix” (Cerón‐Rojas & Crossa, 2018). Vector $\beta_{E_{j}}=Fb_{E_{j}}$ can substitute $b_{E_{j}}$, and in this case, the optimized ESIM index should be written as $I_{E_{j}}={\beta^{\prime }}_{E_{j}}y$.

### 7.3 The maximized ESIM selection response, expected genetic gain per trait, and correlation

Changing **β** = **P** ^−1^ **Cw** (Equation 4) for $b_{E_{1}}$(Equation 38) in Equation 12, the ESIM selection response can be written similarly as the LPSI response. The same is true for the maximized expected genetic gain per trait, while the ESIM correlation is λ_1_, the square root of λ_1_^2^. Cerón‐Rojas and Crossa (2018) showed the ESIM correlation could also be written as Equation 11.

There are some problems associated with ESIM. For example, when matrix **P** is not positive definite and matrix **C** is not positive semidefinite, the eigenvalues of Equation 38 could be negative, and, in such cases, ESIM should not be used to make selections. Nevertheless, Okamoto (1973) showed that when the number of observations was higher than the number of traits, **P** was symmetric and positive definite, and its eigenvalues were different with probability one. In turn, Gentle (2007) showed that when **P** and **C** had the same size, all eigenvalues of **P** ^−1^ **C** existed and were finite. Thus, under the Okamoto (1973) and Gentle (2007) results, ESIM is a good option for making phenotypic selection in breeding programs when it is not possible to obtain vector **w** for the LPSI.

### 7.4 Summary of the ESIM statistical properties

1. The variance of *I_E_* and the covariance between *H_E_* and *I_E_* are the same.
2. The ESIM variance prediction error is $\left(1-\lambda_{1}^{2}\right)\sigma_{H_{E}}^{2}$, where $\sigma_{H_{E}}^{2}$ is the variance of *H_E_*.
3. The relative effectiveness of *I_E_* in predicting *H_E_* is the ratio of $\left(1-\lambda_{1}^{2}\right)\sigma_{H_{E}}^{2}$ over $\sigma_{H_{E}}^{2}$ (i.e., 1 − λ_1_^2^); thus, the greater λ_1_^2^ is, the more effective *I_E_* is in predicting *H_E_*.
4. The mean squared effect of *I_E_* on *H_E_* or the total variance of *H_E_* explained by *I_E_* is $\sigma_{I_{E}}^{2}=\lambda_{1}^{2}\sigma_{H_{E}}^{2}$, and the relative mean squared effect can be measured by $\lambda_{1}^{2}$.

Cerón‐Rojas and Crossa (2018) showed that all the above results are valid for RESIM, PPG‐ESIM, MESIM, and GESIM. Note that the above ESIM properties are very similar to the LPSI described earlier. This is so because the only diference among the LPSI an ESIM is the method to obtain the vector of coefficients.

### 7.5 The constrained eigen selection index method

Similar to the CLPSI (Equation 32), the objective of the predetermined proportional gain ESIM (PPG‐ESIM) is to fix *r* of *t* (*r* < *t*) traits by predicting only the genetic gains of (*t* − *r*) of them. In Equation 32, we gave details associated with the CLPSI that will be used in the subsequent subsections. The statistical bases and objectives of the PPG‐ESIM are the same as those of the CLPSI described in Equation 32. That is, PPG‐ESIM tries to change μ*_j_* to μ*_j_* + *d_j_*, where *d_j_* is a predetermined change in μ*_j_*. In the RESIM, *d_j_* = 0 (*j* = 1, 2, …, *r*), where *r* is the number of predetermined proportional gains.

### 7.6 The PPG‐ESIM vector of coefficients

According to Cerón‐Rojas et al. (2016a), the PPG‐ESIM vector of coefficients that maximizes the PPG‐ESIM selection response and expected genetic gain per trait is

(39) $$\left(K_{E}P^{-1}C-\lambda_{P}^{2}I_{t}\right)b_{P}=0$$

where matrices **K** *_E_* = [**I** *_t_* − **Q** *_E_*] and **Q** *_E_* = **P** ^−1^ **M**(**M**′**P** ^−1^ **M**)**M**′ are the same as those obtained in Equation 32, **I** *_t_* is an identity matrix *t* × *t*, and λ*_P_* ^2^ and **b** *_p_* are the eigenvalue and the eigenvector of matrix **K** *_E_* **P** ^−^ **C**, respectively. When **N** = **U**, **b** *_p_* = **b** *_R_* (the vector of coefficients of RESIM), and when **U**′ is a null matrix, **b** *_p_* = **b** *_E_* (the vector of coefficients of ESIM). That is, PPG‐ESIM is more general than RESIM and ESIM and includes the latter two indices as particular cases.

### 7.7 Relationships among ESIM, PPG‐ESIM, LPSI, and CLPSI

Cerón‐Rojas et al. (2008a, 2016b) described the relationships between the ESIM and PPG‐ESIM vector of coefficients and the LPSI and CLPSI vector of coefficients as follows. When the LPSI and CLPSI vector of coefficients (**β** and **b** *_C_* = **K**β, respectively) are in the space generated by the eigenvectors of Equations 38 and 39, respectively, **β** and **b** *_C_* can be written as

$$\beta =\sum_{j=1}^{t}\alpha_{j}^{*}b_{E_{j}}\;\mathrm{and}\;\;b_{C}=\sum_{j=1}^{t}a_{j}^{*}b_{P_{j}}$$

where α*_j_* * and *a_j_* * are constants that can be estimated by least squares, and $b_{E_{j}}$ and $b_{P_{j}}$ are the eigenvectors of Equations 38 and 39. Based on the Bessel inequality, Cerón‐Rojas et al. (2008a) described a method to determine conditions under which **β** and **b** *_C_* are in the space generated by the eigenvectors of Equations 38 and 39. The above results allowed Cerón‐Rojas et al. (2016b) to find, in the asymptotic context, approximate expectations and variances of the estimators of **β** and **b** *_C_*.

### 7.8 Estimators of the LPSI parameters

Cerón‐Rojas and Crossa (2018) have described the REML method to estimate the LSI parameters. Here, we consider the estimators of the LPSI vector of coefficients, selection response, correlation, and expected genetic gain per trait, as

$$\hat{\beta }={\hat{P}}^{-1}\hat{C}w,\;\hat{R}=k\sqrt{{\hat{\beta }}^{\prime }\hat{P}\hat{\beta }},\;{\hat{\rho }}_{HI}=\frac{\sqrt{{\hat{\beta }}^{\prime }\hat{P}\hat{\beta }}}{\sqrt{w^{\prime }\hat{C}w}},\;\mathrm{and}\;\hat{E}=k\frac{\hat{C}\hat{\beta }}{{\hat{\sigma }}_{I}}$$

respectively, and the estimated LPSI value will be expressed as $\hat{I}={\hat{\beta }}^{\prime }y$. In the above equations, $\hat{P}$(${\hat{P}}^{-1}$) and $\hat{C}$ are REML estimators of the phenotypic (**P**) and genotypic (**C**) covariance matrices, whereas ${\hat{\sigma }}_{I}=\sqrt{{\hat{\beta }}^{\prime }\hat{P}\hat{\beta }}$ is the estimator of the standard deviation of $\hat{I}$, and $\sqrt{w^{\prime }\hat{C}w}$ is the estimator of the standard deviation of *H*.

The REML method is based on projecting the data into a subspace free of fixed effects and maximizing the likelihood function in this subspace (Blasco, 2001). Thus, because $\hat{P}$ (${\hat{P}}^{-1}$) and $\hat{C}$ are REML estimators, by the invariance property of maximum likelihood estimators (Rencher & Schaalje, 2008), $\hat{\beta }$,$\hat{R}$, ${\hat{\rho }}_{HI}$, and $\hat{E}$ are maximum likelihood estimators and they are minimum variance and asymptotic unbiased estimators for **β**, *R*, ρ*_HI_*, and **E** (Cerón‐Rojas & Crossa, 2020b; Muirhead, 2005). Cerón‐Rojas and Crossa (2020b) showed that, in effect, $\hat{R}$ and ${\hat{\rho }}_{HI}$ are asymptotic unbiased estimators and these authors described methods to obtain confidence intervals for the expetations of $\hat{R}$ and ${\hat{\rho }}_{HI}$. The REML estimators can also be used in the CLPSI, ESIM, combined LGSI, multi‐stage LSI, etc.

### 7.9 Numerical example: LPSI vs ESIM

We compare the ESIM efficiency versus LPSI efficiency using a real data set from commercial egg poultry lines obtained from Akbar et al. (1984). The estimated phenotypic ($\hat{P}$) and genetic ($\hat{C}$) covariance matrices among the rate of lay (RL, number of eggs), age at sexual maturity (SM, days), and egg weight (kg), were $\hat{P}=\left[\begin{array}{ccc} 240.57 & -95.62 & 2.07\\ -95.62 & 167.20 & 4.58\\ 2.07 & 4.58 & 22.80 \end{array}\right]$ and $\hat{C}=\left[\begin{array}{ccc} 29.86 & -17.90 & -4.13\\ -17.90 & 18.56 & 1.49\\ -4.13 & 1.49 & 9.24 \end{array}\right]$, respectively. Furthermore, the number of genotypes and the vector of economic weights were *n* = 3,330 and **w**′ = [19.54 −3.56 17.01], respectively, and the selection intensity was 10% (*k* = 1.755) for both indices.

The estimated LPSI vector of coefficients was $\hat{\beta }=w^{\prime }{\hat{P}}^{-1}\hat{C}=\left[\begin{array}{ccc} 1.82 & -1.38 & 3.25 \end{array}\right]$, and the LPSI estimated selection response, expected genetic gain per trait, and correlation was $\hat{R}=k\sqrt{{\hat{\beta }}^{\prime }\hat{P}\hat{\beta }}=74.91$, ${\hat{E}}^{\prime }=k\frac{\hat{\beta^{\prime }}\hat{C}}{\sqrt{{\hat{\beta }}^{\prime }\hat{P}\hat{\beta }}}=\left[\begin{array}{ccc} 2.70 & -2.20 & 0.84 \end{array}\right]$, and ${\hat{\rho }}_{\mathrm{HI}}=\frac{\sqrt{{\hat{\beta }}^{\prime }\hat{P}\hat{\beta }}}{\sqrt{w^{\prime }\hat{C}w}}=0.362$, respectively.

Because in the ESIM context ${{\hat{b}}^{\prime }}_{E_{1}}{\hat{b}}_{E_{1}}=1$, the best way of comparing ESIM results versus LPSI results is when the LPSI coefficient vector is normalized (i.e., when the LPSI coefficient vector is equal to ${\hat{\beta }}^{*}=\hat{\beta }/{\hat{\beta }}^{\prime }\hat{\beta }$ and then ${\hat{\beta }}^{\prime *}{\hat{\beta }}^{*}=1$); however, it can be shown that the normalization process only affects the estimated LPSI selection response because in that case, $\hat{R}=74.91$ is divided by ${\hat{\beta }}^{\prime }\hat{\beta }$. For example, for this data set result, ${\hat{\beta }}^{\prime }\hat{\beta }=15.76$; then, the estimated LPSI selection response using ${\hat{\beta }}^{*}=\hat{\beta }/{\hat{\beta }}^{\prime }\hat{\beta }$ will be $\hat{R}=\frac{74.91}{15.74}=4.75$, while the rest of the estimated LPSI parameters will be the same. When $0<{\hat{\beta }}^{\prime }\hat{\beta }<1$ and $1<\hat{R}$, the values of $\hat{R}$ will increase, but when $1<{\hat{\beta }}^{\prime }\hat{\beta }$, the values of $\hat{R}$ will decrease, as in this example. The product ${\hat{\beta }}^{\prime }\hat{\beta }$ does not affect ${\hat{\rho }}_{HI}$ because it is invariant to scale change. Also, ${\hat{\beta }}^{\prime }\hat{\beta }$ does not affect $\hat{E}$ because ${\hat{\beta }}^{\prime }\hat{\beta }$ appears in the numerator and denominator of both estimated parameters.

In ESIM, the sign and proportion of the expected genetic gain values for traits RL, SM, and egg weight should be according to the breeder's interest. For example, if the breeder's interest is that the expected genetic gain per trait for RL be positive and negative for SM, the sign and proportion of the values of the first eigenvector should be modified using a linear combination of ${\hat{b}}_{E_{1}}$ as described earlier (i.e., ${\hat{\beta }}_{E_{1}}=F{\hat{b}}_{E_{1}}$) in order to achieve expected genetic gain per trait values in RL and SM.

The information needed to obtain the estimated ESIM parameters is matrix ${\hat{P}}^{-1}\hat{C}$. We need to find the eigenvalues and eigenvectors of the equation $\left[\left({\hat{P}}^{-1}\hat{C}\right){\left({\hat{P}}^{-1}\hat{C}\right)}^{\prime }-{\hat{\mu }}_{j}I\right]{\hat{b}}_{E_{j}}=0$, where ${\hat{\mu }}_{j}={\hat{\lambda }}_{E_{j}}^{4}$(Cerón‐Rojas & Crossa, 2018). In this case, ${\hat{\mu }}_{1}={\hat{\lambda }}_{E_{1}}^{4}=0.2115$, ${\hat{\lambda }}_{E_{1}}^{2}=0.4599$ and the estimated ESIM correlation was ${\hat{\lambda }}_{E_{1}}=0.6782$. The estimated ESIM eigenvector of coefficients is ${{\hat{b}}^{\prime }}_{E_{1}}=\left[\begin{array}{ccc} -0.1701 & 0.0259 & 0.9851 \end{array}\right]$, and the estimated ESIM index can be constructed as

$${\hat{I}}_{E}=-0.170RL+0.026SM+0.985EW$$

The estimated ESIM selection response and expected genetic gain per trait were ${\hat{R}}_{E}=1.755\sqrt{{\hat{b^{\prime }}}_{E_{1}}\hat{P}{\hat{b}}_{E_{1}}}=9.54$ and ${{\hat{E}}^{\prime }}_{E}=1.755\frac{{{\hat{b}}^{\prime }}_{E_{1}}\hat{C}}{\sqrt{{\hat{b^{\prime }}}_{E_{1}}\hat{P}{\hat{b}}_{E_{1}}}}=\left[\begin{array}{ccc} -3.10 & 1.61 & 3.18 \end{array}\right]$, respectively. Because the estimated LPSI selection response was $\hat{R}=\left(74.91/15.74\right)=4.75$, the estimated ESIM selection response was higher than the estimated LPSI response for this data set.

Now suppose that the breeder's interest is to increase RL and decrease SM; then ${\hat{E}}^{\prime }$ is a good result but ${{\hat{E}}^{\prime }}_{E}$ is wrong. We can change the sign and proportion of ${{\hat{E}}^{\prime }}_{E}$ by transforming ${\hat{b}}_{E_{1}}$ as ${\hat{\beta }}_{E_{1}}=F{\hat{b}}_{E_{1}}$ using a convenient matrix **F** such as

$F=\left[\begin{array}{ccc} -9 & 0 & 0\\ 0 & 1 & 0\\ 0 & 0 & 1 \end{array}\right].$

In such a case, ${{\hat{\beta }}^{\prime }}_{E_{1}}={{\hat{b}}^{\prime }}_{E_{1}}F=\left[\begin{array}{ccc} 1.531 & 0.026 & 0.981 \end{array}\right]$, ${\hat{R}}_{E}=1.755\sqrt{{\hat{\beta }}^{\prime }\hat{P}\hat{\beta }}=42.44$, and ${{\hat{E}}^{\prime }}_{E}=1.755\left(\hat{\beta^{\prime }}\hat{C}\right)/\left(\sqrt{\hat{\beta^{\prime }}\hat{P}\hat{\beta }}\right)=\left[\begin{array}{ccc} 2.990 & -1.85 & 0.205 \end{array}\right]$. However, vector ${{\hat{\beta }}^{\prime }}_{E_{1}}$ has not been normalized. To normalize ${{\hat{\beta }}^{\prime }}_{E_{1}}$, we need to divide it by ${{\hat{\beta }}^{\prime }}_{E_{1}}{\hat{\beta }}_{E_{1}}=3.314$, but ${{\hat{\beta }}^{\prime }}_{E_{1}}{\hat{\beta }}_{E_{1}}$ should only affect ${\hat{R}}_{E}=42.44$, which should be divided by 3.314; that is, ${\hat{R}}_{E}=42.44/3.314=12.806$. According to the theory of similar matrices (Harville, 2008), the estimated maximized ESIM correlation (${\hat{\lambda }}_{E_{1}}=0.6782$) should not be affected by matrix **F**.

### 7.10 RIndSel

**RIndSel** is a powerful freely available Java software useful for estimating the LPSI, LGSI, CLPSI, ESIM, etc., parameters and selecting individual candidates as parents for the next selection cycle. It is available in the CIMMYT (for its Spanish acronym, or International Maize and Wheat Improvement Center) **RIndSel *Selection indices for plant breeding*** repository at https://data.cimmyt.org/dataverse/root?q=rindsel. **RIndSel** is compatible with Windows XP, 7, 8, and 10 and can be installed on 32‐bit and 64‐bit computers. To input the data information, the user must create an Excel file (saved as a comma‐delimited “.csv”) with the data information to be analyzed, from where the software reads that information and estimates the index parameters.

In the linear mixed model context (Searle et al., 2006), **RIndSel** uses REML and two experimental designs (a randomized complete block design and a lattice or alpha lattice design) to estimate the phenotypic and genotypic covariance matrices. This software presents the results of the estimated index parameters in two Excel files (e.g., *alloutSmith*.csv and *outSmith*.csv, where, in this case, “*Smith”* denotes the LPSI used for the analysis) and one text file (*outextSmith.txt*). File “*alloutSmith*.csv” contains all the trait adjusted means for all genotypes, and the estimated index values, whereas file “*outSmith*.csv” contains the genotypes and the trait values selected with the index values after these have been ranked. File “*outextSmith.txt*” contains all information associated with the estimated index parameters such as the estimated phenotypic and genotypic covariance matrices, the estimated selection response, etc. A complete user manual with instructions on how to use **RIndSel** in plant breeding is available at https://data.cimmyt.org/dataset.xhtml?persistentId=hdl:11529/10854.

### 7.11 Summary of the main results of this review

In this manuscript, we have summarized and expanded upon the results of the following authors:

1. Smith (1936) established the LPSI theory on which all LSIs described in this work are based.
2. Cochran (1951) was the first to indicate that the LPSI is the BLP of the net genetic merit and was the first to develop the two‐stage optimum LPSI.
3. Kempthorne and Nordskog (1959) were the first to develop the RLPSI.
4. Young (1964) combined the LPSI theory with the culling selection method and developed the OMLPSI in the multi‐stage context, from where Cerón‐Rojas et al. (2019b) developed the COMLPSI and Cerón‐Rojas and Crossa (2020a) developed a COMLGSI.
5. Mallard (1972) developed the first optimum constrained LPSI, which allows imposing constraints diferent to zero on the expected genetic gain per trait and includes the RLPSI as a particular case.
6. Xu and Muir (1992) developed the DMLPSI, from where Cerón‐Rojas et al. (2019b) developed a constrained optimum DMLPSI and Cerón‐Rojas and Crossa (2020a) developed a DCMLGSI.
7. Cerón‐Rojas et al. (2008a) and Cerón‐Rojas et al. (2016b) developed the ESIM, RESIM, and PPG‐ESIM in the CCA context.
8. Ceron‐Rojas et al. (2015) and Cerón‐Rojas and Crossa (2019) expanded the unconstrained and constrained LPSI to the genomic selection context and developed the unconstrained and constrained LGSI.

This work complements the classic characterizations of the LSI made by several authors (Bulmer, 1980; Cochran, 1951; Henderson, 1963) and indicates that the LSI theory is a general mathematical‐statistical framework that should allow breeders to confidently use it in plant and animal breeding programs.

## 8 CONCLUSIONS

Populations of plants or animals are sets of individuals that, in turn, are sets of phenotypic characteristics, such as color, weight, height, etc. Thus, when breeders select parents in a breeding program, they select individuals, not single characteristics, and the selection of any set of traits on the individuals changes not only the mean of the individual's unselected traits but also the correlation between selected and unselected traits (Pearson, 1903). Then, in breeding programs, breeders should select parents using LSI because the prediction procedures with univariate models do not contemplate the genetic correlations between traits. In practice, evaluating plants or animals requires several simultaneous traits. For example, breeders studying yield and grain quality register phenotypic data that include yield components (e.g., grain weight or biomass), grain quality (e.g., flavor, shape, color, nutrient content), and resistance to biotic and abiotic stresses (Jia & Jannink, 2012).

We have described the LSI statistical theory assuming that the net genetic merit and the vector of phenotypic values have multivariate normal distribution. Under this assumption, and when the phenotypic and genotypic covariance matrices are known, the LSIs are particular cases of the BLP theory. Furthermore, we found that regardless of the LSI characteristics (single‐stage or multi‐stage, unconstrained or constrained, etc.), the LSI parameters (selection response, expected genetic gain per trait, and correlation) are the same.

The BLP theory provides the mathematical‐statistical framework to develop the LSI theory. Thus, the LSI is a particular case or an application of the BLP to plant and animal breeding selection. This result allows breeders to locate the LSI theory in a general mathematical framework and then confidently use it when making selections. Nevertheless, some problems of the LSI not discussed in this work are associated with the estimation of the LSI parameters. For example, when the phenotypic and genotypic covariance matrices are estimated, the LPSI could not be BLP; indeed, it is not linear in **y** (Searle et al., 2006). Nevertheless, Cerón‐Rojas and Crossa (2020b) found that the LPSI is a good predictor of the net genetic merit including when REML estimates the phenotypic and genotypic covariance matrices. Because of this, breeders should use the LSI theory in plant breeding with confidence.

## AUTHOR CONTRIBUTIONS

J. Jesus Cerón‐Rojas: Conceptualization; Investigation. Jose Crossa: Conceptualization; Investigation.

## CONFLICT OF INTEREST

The authors do not have any conflict of interest.
